# Radiation-Responsive Promoters: Molecular Mechanisms, Screening Strategies, and Translational Applications as Radiation Biomarkers

**DOI:** 10.3390/cimb48040348

**Published:** 2026-03-26

**Authors:** Nanxin Xu, Xin Huang, Pingkun Zhou

**Affiliations:** 1 Hengyang Medical College, University of South China, Hengyang 421001, China; 19916157918@163.com; 2 Department of Radiation Biology, Beijing Key Laboratory for Radiobiology, Beijing Institute of Radiation Medicine, Beijing 100850, China; huangxin.bio@foxmail.com

**Keywords:** radiation-responsive promoters, biological dosimetry, ionizing radiation biomarkers, DNA damage response, epigenetic regulation, synthetic biology, radiogenomics, gene therapy

## Abstract

Radiation-responsive promoters represent a functionally distinct class of transcriptional regulatory elements that translate genotoxic stress signals into quantifiable gene expression outputs. These promoters occupy a unique mechanistic position within the broader radiation biomarker landscape: rather than directly measuring molecular damage products, they report the cellular interpretation of radiation-induced stress through coordinated gene regulatory networks. This review provides a systematic analysis of five major classes of radiation-responsive promoters—microRNA (miRNA) promoters, tRNA-derived small RNA (tsRNA) promoters, acute-phase protein gene promoters, DNA repair gene promoters, and long non-coding RNA (lncRNA) promoters—with emphasis on their regulatory logic, dose-response characteristics, and current evidence for clinical deployment. We further describe four complementary screening strategies: homology-based conservation analysis, functional genomics and transcriptomics, epigenetic modification profiling, and synthetic biology promoter engineering. Applications spanning biosensor development, biological dosimetry, treatment response prediction, and radiation-guided gene therapy are evaluated within a two-track framework that distinguishes biomarker-oriented applications (Track A) from tool-oriented reporter gene systems (Track B). Critical appraisal of current limitations—including insufficient clinical-grade validation, absence of standardized dose-response curves, and reproducibility deficits—is integrated throughout. Future priorities include multi-center prospective validation studies, FAIR-compliant data infrastructure, AI-driven multi-omics integration, and point-of-care detection platforms. Radiation-responsive promoter biology holds significant potential for advancing precision radiotherapy and nuclear emergency medical response, contingent upon systematic closure of the current evidence gap relative to established gold-standard cytogenetic methods.

## 1. Introduction

### 1.1. Diverse Exposure Contexts and Dose Backgrounds of Ionizing Radiation

Ionizing radiation (IR) is a physical agent broadly present in both the natural environment and human technological activities. Exposure contexts encompass multiple dimensions, including outer space, clinical medicine, occupational protection, and environmental contamination. As nuclear technology matures and human spaceflight activities expand, defining radiation types, dose ranges, and biological effects across different exposure scenarios has become essential for radiation protection research and accurate dosimetric assessment.

- Space Radiation and Astronaut Health

During crewed spaceflight missions, astronauts are continuously exposed to a complex radiation field composed of galactic cosmic rays (GCRs) and solar particle events (SPEs). GCRs consist primarily of high-energy protons (~87%), helium nuclei (~12%), and a small proportion of high-charge-and-energy (HZE) heavy ions. The linear energy transfer (LET) of HZE particles can reach several hundred keV/μm, resulting in fundamentally different biological effects compared to low-LET radiation encountered at ground level [1]. During missions to the International Space Station (ISS) in low Earth orbit (LEO), astronauts receive an average effective dose rate of approximately 1–2 mSv/day [2]. For deep space missions such as Mars exploration, where the shielding effect of Earth’s magnetic field is entirely absent, the cumulative effective dose for a single round-trip mission is projected to exceed 1 Sv, substantially increasing the risk of radiation-induced carcinogenesis [3]. The NASA Space Radiation Cancer Risk (NSCR) model currently integrates physical dose measurements, radiation quality factors, and epidemiological data to quantitatively predict the lifetime risk of exposure-induced cancer (REIC) in astronauts; however, this model retains considerable uncertainty in estimating the biological effectiveness of high-LET radiation [4,5].

- Medical Radiation

Medical radiation is the largest source of artificial radiation exposure in contemporary populations, encompassing diagnostic radiology and radiation oncology across dose ranges spanning several orders of magnitude. In diagnostic imaging, the effective dose from conventional radiography is approximately 0.02–1 mSv, while multi-detector computed tomography (CT) scanning delivers effective doses typically in the range of 1–20 mSv, depending on the anatomical region and scanning protocol [6]. Epidemiological evidence indicates that even low-dose diagnostic exposures of this nature exert non-negligible stochastic effects in a large patient population [7]. In cancer radiotherapy, locally prescribed doses within the radiation field routinely reach 20–70 Gy to achieve deterministic cytocidal effects on tumor cells, inevitably inducing radiation bystander effects and abscopal effects in adjacent normal tissues [8]. Accurate assessment of the biological equivalent dose (BED) in the irradiated field and surrounding tissues is critical for protecting organs at risk (OARs) in radiotherapy patients.

- Occupational Exposure and Environmental Radiation

Nuclear industry workers, radiologists, and interventional radiology nurses face chronic low-dose radiation exposure over extended periods. Dosimetric surveillance data from the International Commission on Radiological Protection (ICRP) indicate that, as interventional radiology procedure volumes continue to increase, cumulative annual doses to the eye lens and extremities of medical personnel are rising, with some practitioners approaching the ICRP-recommended annual lens dose limit of 20 mSv [9]. ICRP Publication 103 establishes an annual effective dose limit for occupational exposure of 20 mSv (averaged over 5 years), adjustable to 100 mSv under emergency conditions [10]. With regard to environmental radiation, radon (222Rn), a naturally occurring radioactive gas, constitutes the principal source of indoor radiation exposure. WHO assessment data indicate that radon contributes approximately 3–14% of lung cancer cases, with a pronounced synergistic carcinogenic interaction with tobacco smoking [11]. Major nuclear accidents—notably the 1986 Chernobyl accident and the 2011 Fukushima Daiichi nuclear power plant accident—resulted in regional radionuclide releases that generated complex combined internal and external radiation exposures, posing immense challenges for long-term health surveillance of the affected population [12].

In summary, the radiation type (low-LET X-rays/γ-rays versus high-LET heavy ions or neutrons), dose rate (ranging from μSv/h background radiation to Gy/min therapeutic irradiation), and exposure modality (acute versus chronic fractionated irradiation) across different exposure contexts collectively determine the high diversity of biological endpoints observed. Conventional physical dosimeters can record only the environmental absorbed dose and are unable to reflect individual biological sensitivity, tissue repair kinetics, or complex cellular signaling responses. Consequently, developing precise biological dosimeters that integrate physical dose information with individual biological responses—combining high sensitivity with real-time capability—has become a central priority in radiation biology research.

### 1.2. Overview of Existing Radiation Biomarker Systems

Cytogenetic aberration analysis remains the internationally accepted reference standard for radiation biological dosimetry. Its core principle involves the quantitative enumeration of structural or numerical chromosomal abnormalities induced by ionizing radiation.

#### 1.2.1. Cytogenetic Biomarkers

Dicentric chromosomes are the most classical radiation-specific cytogenetic endpoint, recommended by the International Atomic Energy Agency (IAEA) as the primary biological dosimetry method for acute accidental exposures [13,14]. Their formation mechanism involves the erroneous joining of two chromosomal double-strand break (DSB) ends from two separate chromosomes, generating an abnormal chromosome bearing two centromeres. The yield of dicentric aberrations follows a linear-quadratic dose-response relationship: Y = c + αD + βD^2^, where c represents the spontaneous background rate and α and β are the linear and quadratic coefficients, respectively. This method achieves reliable dosimetric estimates over a range of approximately 0.1–5 Gy for acute whole-body irradiation and is an indispensable diagnostic tool in nuclear emergency medicine [15].

Chromosomal translocations are detected by fluorescence in situ hybridization (FISH). Unlike unstable aberrations such as dicentrics, translocations are stable aberrations transmitted reliably to daughter cells through successive cell divisions, remaining detectable for years to decades after exposure. This characteristic makes them ideal markers for retrospective dosimetry, particularly for historical dose reconstruction in chronically low-dose-exposed populations [16].

Micronuclei (MN) are small nuclear structures formed from lagging chromosomes or chromosomal fragments that fail to incorporate into the main nucleus during late mitosis. The cytokinesis-block micronucleus (CBMN) assay is widely employed due to its operational simplicity, compatibility with automated image analysis, and suitability for large-scale high-throughput screening [17]. The development of automated digital imaging platforms has demonstrated its particular advantage for rapid population-level triage in the early phase of nuclear accidents.

#### 1.2.2. Molecular and Protein-Level Biomarkers

With advances in molecular biology, several rapid molecular biomarkers reflecting radiation-induced immediate damage and oxidative stress have attracted considerable attention.

γ-H2AX foci: When a DSB occurs, histone H2AX within a region spanning hundreds of kilobases flanking the break site undergoes rapid phosphorylation at serine-139 (Ser-139), forming phosphorylated H2AX (γ-H2AX) that assembles into discrete foci detectable by immunofluorescence microscopy. γ-H2AX foci serve as cytological markers of DSB repair kinetics rather than as direct quantitative indicators of absolute DSB number per se; they typically peak at 0.5–2 h post-irradiation and subsequently decline over 24 h as DNA repair proceeds [18]. This indicator demonstrates relatively high sensitivity in the dose range of 10–200 mGy and enables dynamic tracking of damage and repair at the single-cell level; however, its signal-to-noise ratio deteriorates significantly below 50–100 mGy, representing its primary application limitation [19].

8-Hydroxy-2′-deoxyguanosine (8-OHdG): Reactive oxygen species (ROS) generated by ionizing radiation oxidize the guanine base of DNA, producing 8-OHdG. This oxidative damage product can be quantified in urine or tissue by high-performance liquid chromatography-tandem mass spectrometry (HPLC-MS/MS) or enzyme-linked immunosorbent assay (ELISA) and is a recognized indicator of radiation-induced oxidative nucleic acid damage [20].

Comet assay: Single-cell gel electrophoresis detects single- and double-strand breaks at the single-cell level with high sensitivity; however, the degree of methodological standardization requires improvement, and the technique cannot readily distinguish radiation-induced from endogenous DNA damage [21].

#### 1.2.3. Limitations of the Existing Biomarker Framework

Despite their important applications in radiation dose assessment, the existing biomarker framework exhibits several fundamental limitations that constrain widespread adoption in complex exposure scenarios.

First, insufficient timeliness: dicentric chromosome analysis requires 72 h ex vivo culture of peripheral blood lymphocytes and preparation of metaphase chromosomes, precluding rapid population-level triage (≤24 h) in nuclear emergency settings [13]. Second, limited low-dose sensitivity: γ-H2AX foci detection below 100 mGy is substantially confounded by background foci arising from endogenous DNA damage, causing rapid signal-to-noise ratio deterioration and significantly reduced quantitative accuracy [19]. Third, inter-individual variability and confounding factors: non-radiation factors, including age, smoking habit, chronic inflammation, and pre-existing disease, can substantially influence spontaneous micronucleus rates, background translocation frequencies, and basal 8-OHdG concentrations, resulting in large inter-individual baseline differences that seriously compromise the accurate establishment of dose-response relationships [22]. Fourth, insufficient real-time dynamic monitoring capability: existing endpoint biomarkers fundamentally reflect the damage status at specific time points and cannot achieve continuous real-time tracking of radiation biological effects in living systems.

Confronting the inherent tension between “real-time responsiveness” and “signal stability” that characterizes traditional biomarkers, reporter gene systems driven by radiation-responsive promoters (RRPs) present distinctive technical potential. By selecting transcriptional regulatory sequences exhibiting high radiation sensitivity and dose-dependence, these systems can directly convert radiation-induced gene expression signals into visualizable, quantifiable optical or biochemical outputs at the living-cell level, potentially overcoming the inherent delays and invasiveness of traditional methods. This research direction constitutes the core scientific focus of the present review.

### 1.3. Fundamentals of Gene Expression Regulation: Promoters and Transcription Factors

Understanding the operating principles of radiation-responsive promoters requires systematic mastery of the basic mechanisms governing eukaryotic gene expression. The radiation-induced cellular stress response is ultimately reflected in the dynamic remodeling of specific transcriptomes, and the molecular core of this process resides in the precise recognition of and dynamic interaction between transcription factors (TFs), promoters, and cis-regulatory elements (CREs).

#### 1.3.1. Promoter Definition and Hierarchical Architecture

A promoter is a DNA regulatory sequence located near the transcription start site (TSS) of a gene. Its core function is to recruit RNA polymerase and associated transcription factors to precisely initiate and regulate transcription of the downstream gene [23]. Eukaryotic promoters exhibit a highly stratified, modular architecture.

The core promoter, located within approximately ±40 bp of the TSS, is the minimal functional unit for assembly of the pre-initiation complex (PIC). It recruits RNA polymerase II (RNA Pol II) and general transcription factors (GTFs, including TFIIA, TFIIB, TFIID) to initiate basal transcription. Principal functional sequence motifs of the core promoter include the TATA box (located at −28 to −32 bp upstream of the TSS, recognized by the TBP subunit of TFIID), the Initiator (Inr) element (spanning the TSS, the most prevalent core promoter element), the TFIIB recognition element (BRE), and the downstream promoter element (DPE, located at +28 to +34 bp). The combinatorial arrangement of these elements determines the level of basal transcriptional activity [24].

Proximal control elements typically reside 50–250 bp upstream of the TSS and harbor multiple TF binding sites. By interacting with sequence-specific activators or repressors, these elements confer the gene’s initial responsiveness to specific cellular signals, representing the “first tier of signal integration” [25].

Enhancers serve as distal cis-regulatory sequences that may reside kilobases to hundreds of kilobases upstream or downstream of the TSS. They physically contact promoters in three-dimensional space through chromatin looping, dynamically regulating transcriptional activity in a cell-type-specific manner [26]. The activation state of enhancers can be identified epigenetically by specific histone modifications (e.g., H3K4me1, H3K27ac).

#### 1.3.2. Fundamental Differences Between Prokaryotic and Eukaryotic Promoters

Prokaryotic promoter architecture is highly simplified: the RNA polymerase holoenzyme (core enzyme + σ factor) directly recognizes conserved promoter regions (the −10 Pribnow box and −35 region), with specific σ factors mediating recognition of different functional promoters, typically following a “one σ factor, one gene cluster” operon regulatory logic [27]. Prokaryotic transcription and translation can be coupled within the same cellular compartment, without the complexity of post-transcriptional processing.

Eukaryotic promoters, in contrast, exhibit the following fundamental distinctions. First, combinatorial regulatory logic: cooperative, competitive, or antagonistic interactions among multiple transcription factors at promoter/enhancer regions determine “digital switch” transcriptional behavior, conferring high cell-context specificity. Second, chromatin context constraints: eukaryotic DNA is wound around histone octamers to form nucleosomes; promoter activation often depends on chromatin remodeling complexes (e.g., SWI/SNF) reconfiguring nucleosome occupancy, along with epigenetic activation through histone modifications (H3K4me3, H3K27ac) [28]. Third, nucleocytoplasmic transport barrier: the presence of the nuclear envelope requires eukaryotic transcription factors to undergo nuclear localization signal (NLS)-mediated nuclear import before exerting regulatory functions, a step that itself serves as a regulatory node. Fourth, post-transcriptional processing compatibility: primary transcripts driven by eukaryotic promoters require complex processing, including 5′ capping, splicing, and 3′ polyadenylation; hence, promoter design must also account for transcript processing efficiency and stability.

#### 1.3.3. Mechanisms of Transcription Factor Activation Under Radiation Stress

Upon radiation-induced cellular stress, multiple parallel signal transduction pathways rapidly activate specific transcription factors, thereby driving transcriptional upregulation of radiation-responsive genes. The principal mechanisms include the following:

ATM/ATR kinase cascade: DNA double-strand breaks activate ataxia-telangiectasia mutated (ATM) kinase, which phosphorylates p53 protein (at Ser-15 and Ser-20), preventing MDM2-mediated ubiquitin-dependent proteasomal degradation and causing p53 to accumulate and translocate to the nucleus, where it binds p53 response elements (p53REs) in target gene promoters (e.g., CDKN1A, GADD45A), driving transcriptional activation of cell cycle arrest and DNA repair programs [29].

NF-κB pathway: ROS and DNA damage signals induced by IR activate the IκB kinase (IKK) complex, causing phosphorylation and proteasomal degradation of the NF-κB inhibitory protein IκB, releasing NF-κB dimers (primarily p65/p50) for nuclear translocation and binding to κB sites in target gene promoters, driving transcriptional activation of inflammatory cytokines, survival genes, and antioxidant genes [30].

AP-1 (Fos/Jun) pathway: Radiation-induced MAPK (ERK, JNK, p38) signaling cascades activate AP-1 transcription factor family members, which interact with CArG boxes (CC[A/T]6GG) and AP-1 binding sites (TRE/TPA-responsive elements) in target gene promoters, thereby participating in the regulation of cell survival, apoptosis, and inflammatory responses [31].

In summary, the functional core of radiation-responsive promoters resides in their constituent specific cis-responsive elements (CREs). These elements serve as “molecular interfaces” between upstream signal transduction pathways and downstream transcriptional outputs, determining the sensitivity, dose-linear dynamic range, and temporal kinetics of the promoter’s response to radiation stimuli. Systematic screening and functional dissection of these promoter elements constitutes both a theoretical requirement for deepening foundational understanding in radiation biology and the core technical foundation for developing next-generation precision radiation biodosimeters and radiogenetic therapy tools.

## 2. Comparative Framework: Radiation-Responsive Promoters and Existing Biomarkers

Accurate quantification of radiation-induced biological damage is essential for emergency management, dose reconstruction, and clinical intervention following accidental or occupational exposure. Among the available biomonitoring tools, promoter-based transcriptional markers occupy a mechanistically distinct position: rather than directly measuring damage products, they report the cellular interpretation of radiation stress through gene regulatory networks. This section establishes a systematic classification framework for radiation biomarkers, positions promoter-based markers within the existing monitoring landscape, and clarifies the signal transduction logic by which radiation-responsive promoters convert genotoxic signals into measurable transcriptional outputs.

### 2.1. Classification Framework for Radiation Biomarkers

**Figure 1:**
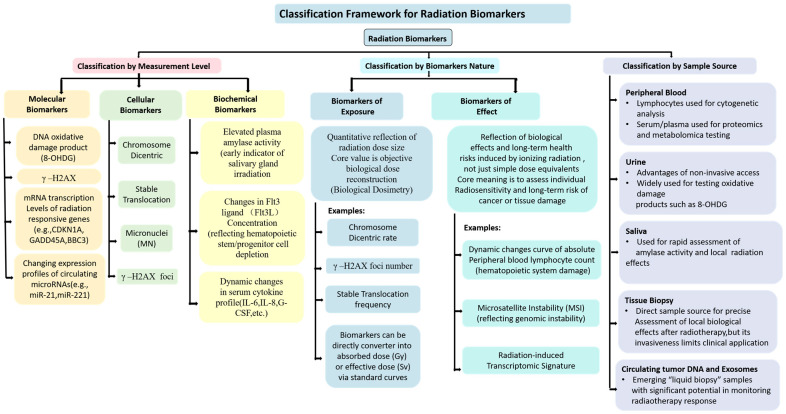
Radiation biomarker system classification diagram.

The systematic classification of radiation biomarkers provides the logical foundation for evaluating the applicability of different detection strategies. Based on three dimensions—measurement level, marker nature, and detection sample source—a clear classification framework can be established for the existing radiation biomarker system (Figure 1).

#### 2.1.1. Classification by Measurement Level

Molecular biomarkers directly reflect chemical damage or gene expression changes induced by ionizing radiation at the nucleic acid, protein, or metabolite level. Representative indicators include the DNA oxidative damage product 8-OHdG, phosphorylated histone H2AX (γ-H2AX), mRNA transcript levels of radiation-responsive genes (e.g., CDKN1A, GADD45A, BBC3), and expression profile changes in circulating microRNAs (e.g., miR-21, miR-221) [32,33]. The core advantage of molecular biomarkers is rapid detection response (minutes to hours), enabling dynamic information acquisition early after damage occurrence; however, some indicators lack radiation specificity and are susceptible to interference from endogenous oxidative stress or other sources of DNA damage.

Cellular biomarkers use morphological or cytogenetic changes in cells as detection endpoints, encompassing dicentric chromosomes, stable translocations, micronuclei, and γ-H2AX immunofluorescence foci [13]. Cellular biomarkers generally require ex vivo culture of peripheral blood lymphocytes; their radiation specificity is relatively strong, and they represent the internationally recognized gold standard for biological dosimetry. However, their principal limitation is the prolonged assay turnaround time (48–72 h).

Biochemical biomarkers target radiation-induced metabolic pathway alterations or protein modifications, including elevated plasma amylase activity (an early indicator of salivary gland irradiation), changes in Flt3 ligand (Flt3L) concentration (reflecting hematopoietic progenitor cell depletion), and dynamic alterations in serum cytokine profiles (IL-6, IL-8, G-CSF) [34,35]. Biochemical biomarkers offer easily accessible sample types (serum/plasma), making them suitable for large-scale screening; however, most indicators have a relatively narrow dose-response linear range and are substantially influenced by non-radiation factors such as inflammation and infection.

#### 2.1.2. Classification by Biomarker Nature

Exposure biomarkers aim to quantitatively reflect the radiation dose received by the organism, with the core value residing in objective biological dose reconstruction. Dicentric chromosome frequency, γ-H2AX foci count, and stable translocation frequency all belong to this category; their results can be directly converted to absorbed dose (Gy) or effective dose (Sv) through standard calibration curves [36]. The value of exposure biomarkers depends on the establishment of precise dose-response relationships and adequate correction for physical parameters, including dose rate, radiation quality (LET), and irradiation modality (uniform/non-uniform).

Effective biomarkers reflect the biological effects and long-term health risks induced by IR, rather than simple dose equivalents. Their core value lies in assessing individual radiosensitivity and long-term cancer or tissue injury risk, such as the dynamic profile of absolute peripheral blood lymphocyte counts reflecting hematopoietic injury, microsatellite instability (MSI) reflecting genomic instability, and radiation-induced transcriptomic signatures [37]. Notably, some markers (e.g., γ-H2AX foci) simultaneously possess attributes of both exposure and effect biomarkers: peak foci number reflects the immediate DSB burden (exposure indicator), while residual foci post-repair correlate with repair capacity and long-term genotoxic risk (effect indicator) [19].

#### 2.1.3. Classification by Detection Sample Source

Different biological samples exhibit significant differences in accessibility, marker stability, and detection sensitivity (Table 1). Peripheral blood constitutes the primary detection matrix: lymphocytes for cytogenetic analysis and serum/plasma for proteomics and metabolomics. Urine, collected non-invasively, is widely used to detect oxidative damage products such as 8-OHdG. Saliva can be used for rapid assessment of amylase activity and local radiation effects. Tissue biopsy provides the most direct sample source for precise assessment of local biological effects post-radiotherapy, though its invasive nature limits clinical applicability. In recent years, circulating tumor DNA (ctDNA) and exosomes, as novel “liquid biopsy” matrices, have demonstrated significant potential in monitoring radiotherapy responses [38].

### 2.2. Distinctive Advantages and Limitations of Promoter-Based Biomarkers

The promoter-based biomarker strategy—employing radiation-responsive promoters to drive reporter genes—represents an emerging paradigm in radiation detection technology. Unlike traditional biomarkers, this approach directly couples radiation-induced transcription signals to detectable optical or biochemical reporter outputs.

#### 2.2.1. Detection Timeliness

The primary constraint of traditional cytogenetic methods is their inherent time delay: dicentric chromosome analysis requires peripheral blood lymphocytes to complete a full cell culture cycle (typically 48–72 h) before metaphase chromosomes can be prepared for microscopic analysis, severely limiting availability during the early rapid triage phase of nuclear emergencies. γ-H2AX foci analysis can reach its detection window within 1–2 h post-irradiation, but the signal decays rapidly within 24 h as DNA repair progresses, imposing strict dependence on an optimal time window [19].

By contrast, detection systems employing radiation-responsive gene promoters (e.g., CDKN1A, GADD45A) to drive reporter genes such as luciferase or GFP can generate detectable fluorescence or luminescence signals within 2–6 h of radiation exposure. The temporal signal-integration property of this system reduces dependence on a narrow post-irradiation time window, in contrast to γ-H2AX foci analysis [43]. Furthermore, reporter proteins engineered with rapid degradation tags (e.g., PEST sequences or DHFR degrons) enable real-time dynamic tracking of promoter activity rather than merely reflecting cumulative effects [44,45].

#### 2.2.2. Low-Dose Detection Sensitivity

Low-dose radiation detection (<100 mGy) represents a shared challenge across the existing biomarker framework.γ-H2AX foci detection below 50–100 mGy is confounded by background foci from endogenous DNA damage in normal cells, causing rapid signal-to-noise ratio deterioration [46]. Dicentric analysis in the low-dose range (<200 mGy) requires analysis of large numbers of cells (≥500) to obtain statistically meaningful estimates, as aberration frequencies approach spontaneous background rates [47].

Promoter-driven reporter systems offer a higher signal amplification ratio: a single activated promoter drives continuous mRNA transcription, and each transcript directs translation of multiple reporter protein copies, cascading from a single DNA damage event to an abundant reporter signal. Studies have demonstrated that optimized luciferase reporter systems can detect signals significantly above background at doses as low as 25–50 mGy, with a detection threshold superior to some traditional methods [43]. However, full realization of this advantage depends critically on careful optimization of the promoter sequence (e.g., multi-copy responsive element arrays), efficient reporter gene translation, and engineered control of near-zero basal transcriptional activity.

#### 2.2.3. Operational Simplicity and Automation Potential

Dicentric analysis requires highly specialized cytogeneticists for manual microscopic scoring; although automated digital imaging systems (e.g., the Metafer platform) have improved throughput to some extent, they cannot fully replace expert manual review [48]. FISH-based translocation detection has a complex workflow with a high technical threshold for probe hybridization and karyotyping, limiting its deployment at resource-limited institutions.

Promoter reporter gene systems, when combined with standard cell culture techniques, multi-well plate readers, or flow cytometers, can achieve highly automated high-throughput screening (HTS). Cell lines stably integrating reporter gene constructs (e.g., TK6-CDKN1A-Luc reporter cell lines), combined with 96- or 384-well plate formats, have been deployed for large-scale compound screening of radioprotectors and radiosensitizers [49], demonstrating throughput advantages unmatched by traditional cytogenetic methods.

#### 2.2.4. Key Limitations

Despite these distinctive advantages, several limitations warrant recognition.

First, radiation specificity: most cis-responsive elements within radiation-responsive promoters (e.g., p53 binding sites, NF-κB binding sites) are not exclusive to radiation; chemical DNA-damaging agents, hypoxia, heat shock, and other stressors can activate the same transcription factor networks to varying degrees, compromising radiation specificity. Mitigation strategies include combining multiple promoters with distinct activation kinetics to construct “logic gate” reporter circuits or incorporating specific response elements activated exclusively by high-LET radiation [50].

Second, requirement for living cell systems: promoter reporter systems depend on intact cellular transcription machinery and must therefore employ living cells (cell lines or whole animal models) as detection platforms. Direct application to rapid field detection of peripheral blood cell samples is presently not feasible, representing a gap relative to the compatibility of cytogenetic methods with primary blood samples [51].

Third, cell line specificity and inter-individual variation: promoter activity is profoundly influenced by cell type, epigenetic background, and basal transcription factor levels. The dose-response relationship of the same radiation-responsive promoter may differ significantly across cell lines of different tissue origins, limiting cross-cell-type applicability [52].

Fourth, signal kinetics interpretation complexity: the steady-state signal of a reporter protein is jointly determined by promoter activity, mRNA stability, translation efficiency, and reporter protein degradation rate. Single-time-point signal measurement cannot accurately reconstruct the dynamic activation history of the promoter; reliable dose estimation requires multi-time-point kinetic data [53].

### 2.3. The Central Role of Promoters in Radiation-Response Signal Transduction

#### 2.3.1. Signal Transduction Chain from DNA Damage to Biomarker Generation

**Figure 2:**
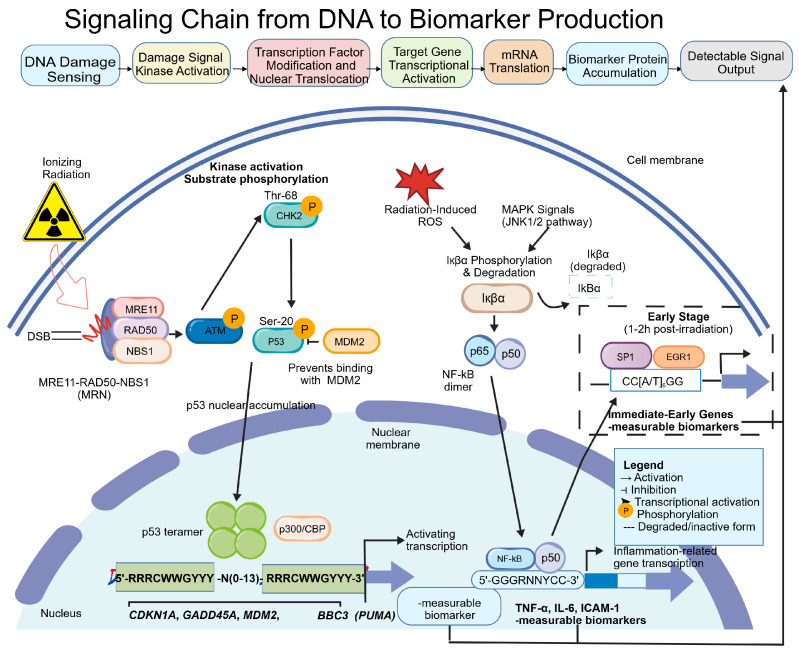
Signaling cascade from ionizing radiation-induced DNA damage to biomarker production. Created with BioGDP.com [54].

The generation of promoter-based biomarkers is a highly integrated molecular signal transduction process, summarized as the following causal chain (Figure 2):

Specifically, IR-induced DSBs are sensed by the MRN complex (MRE11–RAD50–NBS1), activating ATM kinase autophosphorylation and recruitment; ATM propagates the signal downstream by phosphorylating CHK2 (at Thr-68); CHK2 in turn phosphorylates p53 at Ser-20, disrupting the p53–MDM2 interaction and causing stabilizing accumulation of p53 protein [29]. Stabilized p53 protein binds as a tetramer to p53 response elements (5′-RRRCWWGYYY-N(0-13)-RRRCWWGYYY-3′, where R = purine, W = A/T, and Y = pyrimidine) in target gene promoter regions within the nucleus, recruits the transcriptional co-activator p300/CBP, and drives transcriptional activation of target genes including CDKN1A (encoding p21WAF1/CIP1), GADD45A, MDM2, and BBC3 (encoding PUMA) [55].

In parallel, MAPK signaling activated by IR (particularly JNK1/2) and radiation-induced ROS cause IκBα phosphorylation and proteasomal degradation, releasing the NF-κB p65/p50 dimer for nuclear entry and binding to κB consensus sequences (5′-GGGRNNTCC-3′) in target gene promoters, driving transcriptional upregulation of inflammation-related genes (e.g., TNF-α, IL-6, ICAM-1) [30]. In the early phase of the radiation response (1–2 h post-irradiation), transcription factors such as SP1 and EGR1 also participate in the rapid activation of immediate-early genes through binding to CArG boxes (CC[A/T]6GG) [31].

#### 2.3.2. Precise Mechanistic Positioning of γ-H2AX in the Signal Transduction Chain

It is necessary here to provide a precise conceptual definition of the biological significance of γ-H2AX: γ-H2AX is a molecular marker of the cellular response to DSBs, not a direct molecular marker of DSBs themselves. This distinction carries important mechanistic implications. γ-H2AX focus formation is an active chromatin modification process mediated by ATM/ATR/DNA-PKcs kinases; the focus size (~2 Mb chromatin domain) far exceeds the actual DSB site, and there is a temporal lag between focus disappearance and completion of DSB repair; in some cases, foci may resolve prior to complete DSB repair [56]. Additionally, non-radiation sources of DNA damage, such as stalled replication forks and telomere erosion, can also induce γ-H2AX foci, providing a mechanistic explanation at the molecular level for why this marker exhibits limited radiation specificity. Accordingly, when designing radiation-responsive promoter reporter systems using γ-H2AX as the readout signal, these background effects must be incorporated into the signal interpretation framework.

#### 2.3.3. Factors Influencing Promoter Activity

The magnitude of a promoter’s response to radiation stimulation is not determined solely by radiation dose but is rather the dynamic result of multiple intrinsic cellular factors acting in concert.

DNA methylation status: High methylation of CpG islands in promoter regions typically causes occlusion of TF binding sites, substantially suppressing radiation-induced promoter activation. Studies have demonstrated a significant inverse correlation between methylation levels at the CDKN1A promoter region and the magnitude of p53-dependent transcriptional activation in response to radiation [57]. Epigenetic heterogeneity in radiation-responsive promoter methylation across different tumor cell lines is an important epigenetic basis for inter-individual biomarker signal variability.

Chromatin accessibility: Nucleosome occupancy at the promoter region directly determines the physical accessibility for transcription factors. ATAC-seq (assay for transposase-accessible chromatin with high-throughput sequencing) analysis has revealed that radiation treatment can remodel the chromatin open state at specific radiation-responsive gene promoter regions within a short period (1–4 h), creating new open chromatin regions (OCRs) that create physical conditions for TF binding [58]. This epigenetic remodeling process is an important mechanism for rapid activation of radiation-responsive genes and simultaneously implies that the responsiveness of promoter-based markers is profoundly influenced by the basal chromatin state of the cell.

Transcription factor availability and post-translational modification status: The activation efficiency of radiation-responsive promoters ultimately depends on the nuclear concentration of activated TF molecules and their DNA-binding affinity. The degree of p53 stabilization is critically constrained by MDM2 gene amplification status (common in multiple tumor types) and TP53 mutation status: p53 harboring gain-of-function mutations not only lose the ability to bind classical p53 response elements but may also transactivate other target genes by recruiting different co-regulatory factors, fundamentally altering the activation profile of p53-dependent radiation-responsive promoters [59]. Additionally, basal IκBα expression levels and proteasomal activity within the NF-κB pathway have important effects on the efficiency of radiation-induced NF-κB nuclear translocation [30].

#### 2.3.4. The Central Importance of Dose-Response Relationships

The fundamental prerequisite for any biomarker to achieve clinical-grade biological dosimetry is the existence of a reliable, reproducible, quantitative relationship between its signal output and the radiation absorbed dose. An ideal radiation-responsive promoter-based biomarker should satisfy the following dose-response characteristics: monotonic dose-dependence that is linear or described by a well-defined mathematical function within the target dose range; a sufficiently low limit of detection (LOD) to capture low-dose exposure signals; absence of a prominent plateau (saturation) or paradoxical decline at high doses (due to cell death reducing the reporter protein-producing cell population); and an acceptable coefficient of variation (CV ≤ 20%) across different experimental batches and between laboratories [60].

When the dose-response curve of a promoter reporter system meets these requirements, its application value in nuclear emergency dose screening, individualized radiotherapy response monitoring, and space radiation biological dosimetry can be fully realized. For example, a CDKN1A promoter-driven luciferase reporter system has been reported to exhibit a linear dose-response standard curve (R^2^ > 0.95) over the range of 0.1–8 Gy (X-rays), with demonstrated robustness across different cell densities and post-irradiation assay time points, providing important methodological evidence for incorporating promoter-based markers into standardized biological dosimetry protocols [43].

In summary, radiation-responsive promoters occupy a unique signal-integration-node position within the radiation biomarker framework: their activity level represents a comprehensive readout of upstream damage sensing and signal transduction efficiency, while the downstream reporter gene expression they drive provides a quantifiable functional output for biological dosimetry. A deep mechanistic understanding of promoter activity regulation not only facilitates improvement of existing biomarker system performance but also establishes the solid molecular biological foundation for the synthetic biology approaches emphasized below, specifically, the artificial synthesis or engineering modification of radiation-responsive elements to optimize biomarker specificity and sensitivity.

## 3. Primary Types and Mechanisms of Radiation-Responsive Promoters

Radiation-responsive promoters are diverse regulatory nodes that translate genotoxic signals into specific transcriptional programs. This section provides a mechanistically organized account of five major promoter classes: small non-coding RNA promoters (miRNA and tsRNA), protein-coding gene promoters (acute-phase response and DNA repair), and long non-coding RNA promoters. Throughout, a strict distinction is maintained between eukaryotic (human/mammalian) regulatory logic–governed by RNA Pol II, TATA boxes, proximal/distal cis-elements, and chromatin architecture–and prokaryotic (bacterial) systems, which employ sigma factor-dependent promoters fundamentally different in structure and regulatory logic. Where prokaryotic promoters are discussed (Section 5), they are explicitly identified as such.

### 3.1. Small Non-Coding RNA Promoters

Small non-coding RNAs (sncRNAs) have emerged as key mediators of IR-induced post-transcriptional gene regulation. Their expression is driven by radiation-activated transcription factors, making their promoters both mechanistic entry points into the stress response and potential readouts of radiation exposure. Two classes—microRNAs (miRNAs) and tRNA-derived small RNAs (tsRNAs)—are discussed here.

#### 3.1.1. MicroRNA Promoters

- Biogenesis and promoter-level regulation

MicroRNAs are endogenous ~22-nucleotide non-coding RNAs that post-transcriptionally repress gene expression by binding seed sequences in the 3′-untranslated regions (3′-UTRs) of target mRNAs, inducing translational repression or mRNA degradation. miRNA biogenesis is a two-step nuclear-cytoplasmic process entirely dependent on promoter-driven transcription [61]. In the nucleus, the host gene or independent miRNA locus is transcribed by RNA Polymerase II (RNA Pol II) to generate a primary transcript (pri-miRNA), typically several kilobases in length. The Microprocessor complex, comprising the RNase III enzyme Drosha and its co-factor DGCR8, cleaves the pri-miRNA hairpin to release a precursor miRNA (pre-miRNA) of ~60–70 nucleotides. Following nuclear export by Exportin-5 in a Ran-GTP-dependent manner, Dicer cleaves the pre-miRNA in the cytoplasm to generate a mature miRNA duplex, one strand of which is loaded into the RNA-induced silencing complex (RISC). Critically, all regulatory inputs upstream of RISC loading converge on the promoter of the pri-miRNA gene, making the promoter the primary regulatory determinant of miRNA abundance following radiation exposure [61].

- NRF2-mediated regulation of miR-140: a paradigmatic radiation-protective circuit

Among radiation-responsive miRNA promoters, the NRF2-miR-140 axis illustrates how a master redox-sensing transcription factor engages an miRNA promoter to orchestrate radioprotection [62]. NRF2 (Nuclear Factor Erythroid 2-Related Factor 2) is constitutively sequestered in the cytoplasm by the Cullin 3-KEAP1 E3 ubiquitin ligase complex. Radiation-induced reactive oxygen species (ROS) and direct modification of KEAP1 cysteine residues (Cys151, Cys273, Cys288) disrupt KEAP1-NRF2 binding, permitting NRF2 nuclear translocation and binding to antioxidant response elements (AREs) in target gene promoters. Duru and colleagues demonstrated that NRF2 directly binds an ARE within the MIR140 gene promoter, transcriptionally activating miR-140 expression in human lung fibroblasts following 2–8 Gy ionizing radiation [62]. Upregulated miR-140, in turn, suppresses pro-inflammatory targets and enhances cellular radioresistance. It is important to clarify the mechanistic relationship: NRF2 is the transcription factor driving promoter activity, while miR-140 is the transcriptional output conferring radioprotection. The coordinated activity of both–NRF2 as upstream activator and miR-140 as a downstream effector constitutes a coordinated feedforward radioprotective circuit, not merely the parallel transcriptional activity of two independent factors [62].

- Dose-response relationships and temporal kinetics of radiation-responsive miRNAs

The dose-dependent modulation of miRNA expression following IR has been characterized for several radiation-responsive species. Templin et al. profiled miRNA expression in peripheral blood mononuclear cells of radiotherapy patients receiving fractionated doses, identifying consistent upregulation of miR-21, miR-29a, and miR-150 with dose accumulation [63]. Josson et al. demonstrated that miR-21 is upregulated in a dose-dependent manner in prostate cancer cells exposed to 2–8 Gy X-rays, with peak induction (~3-fold) at 24–48 h post-irradiation and return toward baseline by 72 h [64]. miR-34a, a direct transcriptional target of p53, follows p53 activation kinetics: induced within 4–8 h of genotoxic stress at doses sufficient to stabilize p53 (>0.5 Gy) and exhibiting linear dose-proportional induction up to approximately 10 Gy in p53-proficient cell systems [65,66]. Conversely, miR-7 is consistently downregulated following IR in multiple cell types, with nadir expression at 12–24 h [65].

- Advantages and limitations of radiation biomarkers

Serum and plasma miRNAs offer a compelling non-invasive detection matrix: they are packaged within exosomes or bound to Argonaute-2 proteins that confer stability against RNase degradation in biofluids. The resulting diagnostic window spans hours to days post-exposure, complementing the narrow 24 h window of gamma-H2AX foci detection [65]. However, several limitations presently constrain their deployment as primary biodosimetric markers. First, miRNA promoters are structurally heterogeneous. Most miRNAs lack canonical TATA boxes and are controlled by combinations of diverse transcription factor binding sites whose composition varies between cell types and tissues, impeding the design of universally applicable promoter-reporter constructs [61]. Second, miRNA expression is substantially altered by non-radiation conditions, including inflammation, infection, cancer, aging, and metabolic stress, all of which are common confounders in radiation-exposed populations. Third, inter-study reproducibility has been poor due to pre-analytical variability (hemolysis, extraction protocols), absence of standardized reference normalization, and differences in irradiation conditions. Rigorous dose-response calibration and validation in human cohorts remain prerequisites before serum miRNA profiling can attain clinical-grade biodosimetric utility [63,65].

#### 3.1.2. tRNA-Derived Small RNAs (tsRNAs)

- Biogenesis: nucleolytic cleavage of mature tRNA

tRNA-derived small RNAs (tsRNAs) are a heterogeneous class of sncRNAs generated by endonucleolytic cleavage of mature cytoplasmic tRNAs, distinct from miRNA biogenesis and largely independent of the canonical Drosha-Dicer pathway [67,68]. Two major subtypes are recognized. tRNA-derived stress-induced RNAs (tiRNAs, also termed tRNA halves) are approximately 30–40 nucleotides in length and arise from cleavage at the anticodon loop by angiogenin (ANG), a stress-activated member of the RNase A superfamily; ANG is released from ribonuclease inhibitor (RNH1) under oxidative and genotoxic stress conditions, providing a direct mechanistic link to IR exposure [67]. tRNA- derived RNA fragments (tRFs) are smaller (14–30 nucleotides), generated by Dicer or other unidentified nucleases at various cleavage positions (5′-tRF, 3′-tRF, internal tRF). Both tiRNAs and tRFs are ultimately derived from the cleavage of mature tRNAs, whose transcription from Pol III-dependent tRNA gene promoters is subject to genotoxic stress regulation via mechanisms including upstream promoter element (UPE) accessibility and RNA Pol III activity modulation [68].

- Radiation response and dose-dependent expression

tsRNAs have been identified as novel radiation-responsive biomarkers exhibiting distinct spatiotemporal characteristics. Wei et al. performed blood multi-omics profiling of mice exposed to carbon ion, proton, and X-ray irradiation across dose ranges of 0.5–4 Gy and identified 15 tsRNAs demonstrating statistically significant dose-dependent expression changes [69]. All 15 tsRNAs possessed conserved upstream promoter sequences, and their expression alterations were observable in both serum and peripheral blood mononuclear cells (PBMCs), suggesting combined intracellular transcriptional regulation and extracellular release mechanisms [69]. A particularly informative finding concerns tRF-Gly-GCC: Deng et al. demonstrated a rapid and acute decrease in serum tRF-Gly-GCC levels within 30 min following carbon ion irradiation in a murine model, with the magnitude of decline exhibiting a definite dose-response relationship [70]. The mechanism involves tRF-Gly-GCC participation in the regulation of oxidative stress response pathways in radiation-induced lung injury, establishing its relevance not merely as a passive biomarker but as a functional mediator of radiation-induced tissue damage [70].

- Circulating tsRNAs and single-cell sequencing: clarifying the mechanistic relationship

The co-deployment of circulating tsRNA measurements with single-cell RNA sequencing (scRNA-seq) warrants mechanistic clarification, as these methodologies interrogate different biological compartments. Circulating tsRNAs in plasma and serum are predominantly packaged within exosomes; 30–150 nm lipid bilayer vesicles are constitutively released from diverse cell types, the release rate of which increases substantially following genotoxic insult [71]. Circulating tsRNA profiles therefore represent a population-level aggregate signal reflecting the average molecular response of multiple irradiated cell types, weighted by their exosome secretion rate. In contrast, scRNA-seq provides single-cell resolution of the transcriptional landscape—including tsRNA promoter activity in individual cells—enabling identification of cell-type-specific response heterogeneity that is masked in bulk tissue or circulating RNA measurements [69]. The two approaches are therefore complementary: scRNA-seq identifies which cell types within irradiated tissue activate specific tsRNA promoters and contribute most to circulating tsRNA signals, while circulating tsRNA quantification provides a minimally invasive integrated readout suitable for clinical monitoring. Integration of these methodologies—using scRNA-seq to calibrate the cellular origin of circulating tsRNA species—represents a promising but not yet realized research direction [69].

- Advantages and current limitations

tsRNAs offer several properties favorable for radiation biomarker development: non-invasive detection in biofluids, responsiveness within minutes (tRF-Gly-GCC decline at 30 min), and apparent sensitivity to radiation quality (differential response to carbon ions versus X-rays) [69,70]. However, significant challenges remain. The stability of specific tsRNA species in plasma is poorly characterized across pre-analytical conditions. No standardized quantification reference exists. The promoter-level regulatory mechanisms driving tsRNA induction remain largely uncharacterized for most species. Furthermore, the functional consequences of tsRNA level changes—whether they are drivers of radiation response or merely passengers of tRNA metabolic reprogramming—have not been resolved for most species [68].

### 3.2. Protein-Coding Gene Promoters

Promoters regulating protein-coding genes constitute a second major class of radiation-responsive regulatory elements. Unlike sncRNA promoters, they directly control the expression of functional proteins involved in systemic stress responses and intracellular DNA damage repair, providing both measurable biomarker outputs and mechanistic insights into the cellular response to IR.

#### 3.2.1. Acute-Phase Response Protein Promoters

- The acute-phase response as a systemic radiation biomarker

The acute-phase response (APR) is a conserved systemic inflammatory reaction initiated by cytokine signals emanating from sites of tissue damage, including IR-induced injury. Ionizing radiation induces tissue destruction and pathogen-associated molecular pattern (PAMP)-independent sterile inflammation, triggering the release of IL-1beta, IL-6, and TNF-alpha from injured stromal and immune cells. These cytokines reach the liver via the systemic circulation, where they engage hepatocyte surface receptors and activate intracellular signaling cascades that converge on the promoters of acute-phase protein (APP) genes [72]. The resulting upregulation of APP synthesis—including serum amyloid A (SAA), C-reactive protein (CRP), fibrinogen, and interleukin-1 receptor antagonist (IL-1Ra)—constitutes a measurable systemic response to IR exposure accessible through standard serum protein quantification [73,74].

- Transcription factor regulatory networks at APP promoters

APP promoter regulation involves cooperative binding of at least three major transcription factors [73,75]: (i) NF-kappaB, activated by IL-1beta-induced IKK-dependent IkappaB phosphorylation and proteasomal degradation, binds canonical kappaB response elements in SAA, CRP, and IL-6 promoters; (ii) C/EBP isoforms, particularly C/EBPbeta (NF-IL6) and C/EBPdelta, which are rapidly induced by IL-6/STAT3 signaling and bind CCAAT box elements in APP promoters, often in cooperative interaction with NF-kappaB; (iii) STAT3, activated by JAK1/2 kinases downstream of IL-6 receptor engagement, binds STAT-response elements (TGCNN(N)3GAAA) in CRP, SAA, and haptoglobin promoters. This combinatorial regulatory architecture ensures that APP promoters function as integrating signal processors, responding not to a single upstream cue but to the coincidence of multiple cytokine signals proportional to the severity and extent of radiation-induced tissue damage [73,75].

- CBCS enhancement of DNA repair gene promoters

Beyond hepatic APPs, radiation-induced modulation of DNA repair gene promoters by extracellular factors has been demonstrated in a human cellular system. Takaoka et al. showed that the culture supernatant of Clostridium butyricum TO-A (CBCS), a probiotic bacterial strain, significantly enhanced the promoter activities of human DNA repair genes ATM, PARP1, and RB1 in normal human dermal fibroblasts and HEK293 cells, as quantified by luciferase reporter assays [76]. This finding suggests that microbiota-derived metabolites in CBCS may act as epigenetic modulators of DNA repair gene promoter accessibility, although the specific active components and their mechanisms of promoter chromatin remodeling remain to be characterized [76].

- Advantages and limitations of radiation biomarkers

APPs offer the practical advantage of detection in standard clinical chemistry platforms (serum CRP by high-sensitivity immunoturbidimetry; SAA by ELISA) without specialized molecular biology infrastructure. Their dose-response characteristics have been characterized in animal models: SAA and CRP show proportional elevation at whole-body doses above 1 Gy, with peak concentrations at 12–72 h post-exposure [77]. However, a fundamental limitation constrains their deployment as specific radiation biomarkers: APPs are acute-phase reactants induced by any significant tissue trauma, infection, or systemic inflammation, lacking radiation specificity. Their utility is therefore primarily as adjunct indicators confirming systemic injury rather than as primary dosimetric tools.

#### 3.2.2. DNA Repair-Related Gene Promoters

- The p53-CDKN1A(p21) axis: a quantitative dose-sensing circuit

The p53-p21 transcriptional axis represents one of the best-characterized radiation-responsive promoter circuits in eukaryotic cells and serves as a paradigm for quantitative dose-sensing. Upon IR-induced DSB formation, ATM undergoes autophosphorylation at Ser1981 and phosphorylates p53 at Ser15, disrupting the MDM2-p53 interaction and stabilizing p53 through prevention of proteasomal degradation. Subsequent phosphorylation events (Chk2-Ser20; p300/CBP-mediated acetylation at Lys382) confer full transcriptional competency. The CDKN1A (p21/WAF1/CIP1) gene harbors two canonical p53 response elements—each a pair of degenerate 5′-RRRCWWGYYY-3′ decamers separated by 13 base pairs—located approximately 1.4 kb and 2.4 kb upstream of the transcription start site; p53 binding to these elements drives robust transcriptional activation [78]. The resulting p21 protein inhibits cyclin-CDK complexes, imposing G1/S cell cycle arrest that enables DNA repair prior to replication.

Quantitative dose-response analysis by Amundson et al. demonstrated that CDKN1A mRNA induction in irradiated human cells follows an approximately linear relationship with dose in the range of 0.1–2 Gy, with a slope of approximately 2- to 4-fold induction per Gy depending on cell type and endpoint timing [79]. At doses exceeding 5 Gy, p53 signaling saturation and the activation of alternative apoptotic programs introduce non-linearity, and CDKN1A induction plateaus or decreases as apoptotic pathways override arrest responses [79]. This well-defined dose range (0.1–5 Gy) positions p21 promoter-driven reporters as particularly relevant for the dose ranges encountered in radiation triage scenarios.

- GADD45 family promoters: AP-1 and NF-kappaB co-regulation

The GADD45 (Growth Arrest and DNA Damage) protein family (GADD45A, GADD45B, GADD45G) functions at the G2/M checkpoint and in nucleotide excision repair. The GADD45A promoter contains a binding site for the Wilms Tumor 1 (WT1) transcription factor and a p53 response element, enabling dual regulation by p53-dependent and -independent pathways [80]. A UV radiation response element (UVRE) within the GADD45A proximal promoter—distinct from the IR-responsive p53 RE—enables transcriptional activation by AP-1 (c-Jun/c-Fos heterodimers) following oxidative stress and non-ionizing radiation. This dual regulatory architecture allows GADD45A to function as a convergence point for multiple genotoxic signals, though it also implies reduced specificity for ionizing radiation relative to the more selective p53-CDKN1A axis [80].

- BEND4: a newly identified epigenetically regulated DNA repair gene

BEND4 (BEN Domain Containing 4) has recently been identified as a novel DNA damage response gene with an epigenetically regulated promoter. Yao et al. demonstrated that BEND4 promoter hypermethylation is a frequent event in pancreatic ductal adenocarcinoma, resulting in transcriptional silencing of BEND4-mediated DNA repair functions [81]. Using promoter methylation profiling and CRISPR-based functional validation, the authors established that BEND4 silencing by promoter hypermethylation renders pancreatic cancer cells synthetically lethal to ATM kinase inhibitors, providing a mechanistic rationale for ATM inhibitor deployment in BEND4-methylated tumors, an example of directly translating promoter epigenetics into a predictive biomarker for targeted radiosensitization therapy [81]. The biological function of BEND4 in the DSB repair pathway choice (NHEJ versus HR) is under active investigation.

- Inter-individual variability and confounding factors: evidence

A critical translational challenge for DNA repair gene promoters as biodosimetric markers is the substantial inter-individual variability in baseline expression and radiation-induced induction magnitude. This variability arises from multiple sources. Genetic polymorphisms in promoter regulatory regions and in DNA repair genes themselves contribute functionally significant differences: the XRCC1 Arg399Gln polymorphism (rs25487), for example, alters the efficiency of base excision repair, modifying the upstream signaling context within which DNA repair gene promoters are activated [82]. A comprehensive meta-analysis by Barnett et al. identified numerous germline variants in radiation response genes—including promoter-region variants in TGFB1, ATM, and XRCC1—that are significantly associated with inter-patient differences in normal tissue radiation toxicity, with effect sizes sufficient to substantially shift individual dose-response curves [83]. Extrinsic confounders include concurrent chemotherapy (many alkylating agents independently activate p53 and GADD45), pre-existing inflammatory states (constitutive NF-kappaB activation in inflamed tissue elevates baseline APP expression), age (progressive decline in homologous recombination repair capacity with age), and sex (sex-hormone-dependent modulation of NF-kappaB activity). These sources of variability mandate individual calibration and multivariate normalization approaches for deployment of DNA repair gene promoter markers in clinical biodosimetry [83].

### 3.3. Long Non-Coding RNA (lncRNA) Promoters

Long non-coding RNAs (lncRNAs), defined as RNA transcripts exceeding 200 nucleotides without protein-coding capacity, constitute a functionally diverse class of regulatory molecules with important roles in IR response. Unlike miRNAs—which primarily regulate gene expression post-transcriptionally in the cytoplasm—lncRNAs predominantly function in the nucleus, acting directly at the interface of chromatin architecture and transcriptional regulation. Their expression is governed by promoters regulated by radiation-activated transcription factors, and their mechanistic diversity gives rise to distinct and sometimes opposing roles in cellular radiation sensitivity [84].

#### 3.3.1. Mechanisms of lncRNA-Mediated Radiation Response

(1) Chromatin remodeling: histone modification scaffolding

Many lncRNAs function as molecular scaffolds that recruit histone-modifying enzyme complexes to specific genomic loci. Radiation-induced lncRNAs interact with components of the Polycomb Repressive Complex 2 (PRC2, including EZH2 and SUZ12) and Trithorax group complexes to deposit repressive H3K27me3 or activating H3K4me3 marks at target gene promoters, respectively. This mechanism enables radiation-induced lncRNAs to indirectly regulate the promoter landscape of downstream genes without themselves encoding protein products [84].

(2) Transcriptional regulation: transcription factor complex scaffolding

A subset of lncRNAs function as transcriptional co-activators or co-repressors by providing structural scaffolds that stabilize or displace transcription factor complexes at promoters. Radiation-responsive lncRNAs may interact with components of the Mediator complex, general transcription factors, or sequence-specific TFs such as p53, altering the kinetics and magnitude of p53-dependent transcription at target gene promoters following DSB-induced p53 stabilization [84,85].

(3) Recruitment of DNA repair proteins to DSB sites

A mechanistically distinct and clinically significant function of radiation-induced lncRNAs is the direct recruitment of DNA repair proteins to DSB sites. Zhang et al. identified LINP1 (lncRNA in non-homologous end-joining pathway 1) as a nuclear lncRNA whose expression is upregulated in triple-negative breast cancer cells and whose promoter is activated by epidermal growth factor receptor (EGFR) signaling [86]. LINP1 directly binds the Ku70-Ku80 (XRCC6-XRCC5) heterodimer—the primary sensor of DSB ends in the NHEJ repair pathway—through a specific secondary structure domain and simultaneously binds DNA-PKcs, thereby stabilizing the Ku70-Ku80-DNA-PKcs ternary complex at DSB ends and enhancing NHEJ repair efficiency. Importantly, LINP1 expression inversely correlates with p53 activity: p53 transcriptionally suppresses LINP1 expression by binding a p53 response element in the LINP1 promoter, providing a mechanistic explanation for the observation that p53-deficient tumors exhibit higher LINP1 levels and enhanced radioresistance [86]. The p53-LINP1 antagonism represents a direct promoter-level regulatory link between the canonical DSB sensing cascade and NHEJ repair capacity.

#### 3.3.2. Representative lncRNA Promoters in Radiation Response

- ZFAS1: promoter-driven radioresistance in nasopharyngeal carcinoma

ZFAS1 (Zinc Finger Antisense 1) is an lncRNA upregulated in nasopharyngeal carcinoma (NPC) that promotes radioresistance through a ceRNA (competing endogenous RNA) mechanism. Peng et al. demonstrated that ZFAS1 functions as a molecular sponge for hsa-miR-7-5p, sequestering miR-7-5p and preventing its targeting of ENO2 (Enolase 2) mRNA [87]. Relief of miR-7-5p-mediated repression leads to ENO2 upregulation, which enhances glycolytic flux and promotes radiation resistance in NPC cells. The promoter of ZFAS1 in NPC has not been fully characterized at the transcription factor level; its transcriptional regulation likely involves multiple tumor microenvironment signals. It is important to note that claims regarding ZFAS1-mediated G1/S cell cycle arrest and apoptosis induction derive from distinct mechanistic studies in different cancer models and should not be generalized to its function in NPC radioresistance, where the predominant mechanism is ENO2 upregulation via miR-7-5p sponging [87].

- VIM-AS1: promoter polymorphism as a modifier of radiation-induced fibrosis

VIM-AS1 (Vimentin Antisense 1) is a nuclear lncRNA whose promoter region harbors functionally significant genetic variants that modulate radiation-induced normal tissue fibrosis. Schlieve et al. established VIM-AS1 as a key regulator of radiation-induced fibrosis in breast tissue following radiotherapy [88]. Mechanistically, VIM-AS1 functions within a regulatory axis involving TGFB1 (transforming growth factor beta 1) and VIM (vimentin): radiation-activated TGFB1 signaling transcriptionally upregulates VIM-AS1 expression, and VIM-AS1 in turn promotes VIM expression by modulating the epigenetic landscape of the VIM promoter. Promoter-region single-nucleotide polymorphisms (SNPs) in the VIM-AS1 locus alter the efficiency of this TGFB1-driven transcriptional activation, providing a genetic basis for inter-individual differences in post-irradiation fibrosis susceptibility [88]. This example illustrates how promoter polymorphisms in lncRNA genes can function as biomarkers of radiation effect (predicting individual toxicity) rather than biomarkers of exposure.

- HAR lncRNAs: clarifying the relationship to radiation biology

Histone acetylation-regulated (HAR) lncRNAs are a transcriptional category identified by chromatin immunoprecipitation sequencing (ChIP-seq) for H3K27ac (an active enhancer/promoter mark). Li et al. constructed a prognostic classifier based on HAR lncRNA expression profiles in lung adenocarcinoma (LUAD), demonstrating a significant correlation between HAR lncRNA expression patterns and both patient survival and immunotherapy response [89]. The relevance of HAR lncRNAs to radiation response requires careful delineation. The correlation between HAR lncRNA signatures and immunotherapy response suggests that these lncRNAs may participate in the regulation of the tumor immune microenvironment, a domain increasingly recognized as critical for the efficacy of radiation-induced immunogenic cell death and radiation-immunotherapy combinations [89]. However, a direct mechanistic link between HAR lncRNA promoter activity and radiation protection or radiation sensitivity has not been established. The hypothesis that HAR lncRNA-derived models could inform radiation protection applications requires independent experimental validation, specifically, functional studies demonstrating lncRNA-dependent modulation of cellular radiation survival, DSB repair, or normal tissue radiation tolerance, none of which has been reported to date [89]. This distinction is critical for accurately representing the current state of evidence.

#### 3.3.3. Potential and Limitations of lncRNA Promoters as Biomarkers

lncRNA promoters offer mechanistic diversity unmatched by miRNA or protein-coding gene promoters: a single radiation-responsive lncRNA can regulate chromatin structure, transcription factor occupancy, and DNA repair pathway choice simultaneously. Their highly tissue-specific expression patterns may, conversely, enable the development of tissue-type-specific radiation biomarkers capable of distinguishing between different sites of radiation exposure, a property not shared by systemic biomarkers such as APPs or cytogenetic assays [84]. However, two major limitations presently preclude broad clinical deployment. First, the tissue-specific expression patterns that confer mechanistic specificity also drastically limit non-invasive detection: unlike miRNAs and tsRNAs, most nuclear lncRNAs are poorly represented in plasma or serum, requiring tissue biopsy or invasive sampling. Second, lncRNA annotation and functional characterization are markedly less advanced than for protein-coding genes: the majority of predicted lncRNA transcripts lack experimental validation of their radiation-responsive promoter regulatory mechanisms, and cross-study reproducibility remains low due to transcript isoform complexity and platform-dependent detection variability [84,85].

### 3.4. Summary: Comparative Overview of Radiation-Responsive Promoter Classes

The five classes of radiation-responsive promoters discussed in this section differ fundamentally in their regulatory logic, detection feasibility, dose-response characteristics, and current evidence maturity. Table 2 provides a structured comparative overview to enable their evaluation as complementary components of a multi-analyte biomarker strategy.

APP, acute-phase protein; APR, acute-phase response; CBMN, cytokinesis-block micronucleus; DSB, double-strand break; NHEJ, non-homologous end-joining; PBL, peripheral blood lymphocyte; qRT-PCR, quantitative reverse transcription PCR; SAA, serum amyloid A; scRNA-seq, single-cell RNA sequencing; SNP, single-nucleotide polymorphism; TF, transcription factor; tsRNA, tRNA-derived small RNA.

This comparative analysis reveals an important landscape insight: no single promoter class currently satisfies all criteria for a standalone clinical radiation biomarker. An optimal multi-analyte strategy would combine the rapid, sensitive cytoplasmic indicators (miRNA, tsRNA) for early post-exposure screening, the well-validated nuclear stress response markers (p21, GADD45A) for quantitative dose assessment in the 0.1–5 Gy range, and the epigenetically stable MGMT methylation platform for predictive profiling in radiotherapy patients. lncRNA-based markers, while mechanistically rich, currently remain most suitable for exploratory research and mechanistic dissection rather than primary clinical biodosimetry.

## 4. Screening Strategies and Methods for Radiation-Responsive Promoters

The systematic screening of radiation-responsive promoters is a core step in constructing radiation exposure biomarker detection frameworks and developing radiation diagnostic and therapeutic tools. Based on research objectives and application context, current screening strategies fall into two complementary tracks: Track A (biomarker-oriented strategy), which aims to identify radiation-responsive regulatory elements from the natural genome for establishing non-invasive exposure detection methods, and Track B (tool-oriented strategy), which employs artificial design and engineering modification to construct highly sensitive reporter gene detection systems for radiation gene therapy and dose-monitoring biosensor development. These two tracks complement each other and together drive the sustained deepening of research in the radiation-responsive promoter field. This section describes four core screening strategies—homology and conservation analysis, functional genomics and transcriptomics, epigenetic modification profiling, and synthetic biology promoter engineering—discussing the technical principles, scope, and limitations of each.

### 4.1. Screening Based on Homology and Conservation Analysis

Cross-species sequence alignment is a classical strategy for identifying functional regulatory elements. Its core logic is that sequences preserved under evolutionary selection pressure typically carry important biological functions. By systematically comparing promoter regions of orthologous genes between humans and model organisms such as mice and rats, researchers can efficiently identify TF binding sites, enhancer modules, and other cis-regulatory elements, focusing screening efforts on conserved sequence segments with high functional credibility [90].

In the identification of radiation-responsive regulatory elements, this strategy has yielded several representative findings. The identification of a DNA damage response element (DDRE) within the GADD45 (growth arrest and DNA-damage-inducible 45) gene promoter is a classic example of this approach. Through deletion mutagenesis and cross-species sequence comparison, researchers precisely mapped a ~50 bp DDRE core sequence within a conserved region shared by human and mouse GADD45 gene promoters, demonstrating that this element mediates GADD45 transcriptional activation in response to UV radiation, ionizing radiation, and various alkylating agents [90,91]. The high degree of inter-species conservation of the DDRE sequence implies a universal DNA damage surveillance function in mammals, establishing an important foundation for using GADD45 and its regulatory elements as radiation-responsive molecular targets.

The discovery of the late DNA damage UV-B responsive element (LDUVB) further extended the application of conservation analysis in radiation biology. LDUVB was initially identified through sequence conservation analysis in plant-related gene promoter regions; this element specifically mediates DNA damage signal responses induced by UVB radiation [92]. It is important to note that the signaling mechanisms underlying UVB radiation responses mediated by LDUVB are fundamentally distinct from those of ionizing radiation (X-rays, γ-rays): UVB responses primarily activate the nucleotide excision repair (NER) pathway and specific photomorphogenesis signaling networks, whereas IR responses depend primarily on ATM/ATR kinase-mediated DSB repair cascades [93]. Accordingly, when attempting to extend LDUVB-type responsive elements to ionizing radiation detection systems, rigorous experimental validation of response specificity is required to avoid false positives caused by signaling pathway cross-activation.

The primary advantage of homology and conservation analysis lies in its strong biological prior; conserved regulatory elements have typically passed the functional filter of prolonged evolutionary selection, conferring high reliability. However, the method also has inherent limitations: rapid evolution of non-coding regions between species may cause important regulatory elements to be overlooked, making results species-dependent; furthermore, the strategy is fundamentally applicable only to discovering naturally conserved elements and lacks discovery power for novel radiation-responsive sequences that are evolutionarily unconserved or generated by recent genomic rearrangements. Future research can address these limitations by integrating high-throughput ChIP-seq with CRISPR-Cas9 functional validation systems to achieve an efficient closed-loop pipeline from candidate sequence identification to functional confirmation [94].

### 4.2. Screening Based on Functional Genomics and Transcriptomics

The rapid development of functional genomics and transcriptomics technologies has enabled researchers to systematically characterize the dynamic regulatory features of radiation-responsive promoters at a genome-wide scale, achieving a fundamental transition from the single-gene, experience-driven traditional research paradigm to a multi-dimensional, data-driven modern approach.

In systematic chromatin-level analysis, ChIP-seq (chromatin immunoprecipitation sequencing) and ATAC-seq (assay for transposase-accessible chromatin with sequencing) are the two most widely applied core technologies. ChIP-seq systematically identifies promoter regions activated or suppressed under radiation conditions through genome-wide, high-resolution mapping of binding profiles for specific transcription factors (e.g., p53, NF-κB, EGR1) or histone modification marks (e.g., H3K4me3) [95]. ATAC-seq exploits the preferential cleavage of open chromatin regions by the Tn5 transposase, enabling rapid quantitative detection of genome-wide chromatin accessibility from low cell numbers (500–50,000 cells), providing an efficient tool for identifying radiation-induced chromatin remodeling events and associated regulatory elements [96]. The combined application of these two technologies can cooperatively characterize the activation state of radiation-responsive promoters from two complementary dimensions—TF occupancy and chromatin openness—substantially improving the precision of candidate target identification.

Differential gene expression analysis based on RNA-seq (RNA sequencing) provides another important pathway for promoter screening. Through systematic comparison of whole-transcriptome differential expression profiles before and after radiation treatment, a set of specifically radiation-responsive genes can be identified, and their promoter regions can then be reverse-engineered to identify functionally relevant cis-regulatory elements. This reverse-inference strategy—from phenotypic transcriptional changes to regulatory sequences—has been effectively validated in multiple radiation biology studies, substantially reducing the initial screening scope for candidate promoters and improving the efficiency of subsequent experimental validation [97].

The emergence of single-cell multi-omics technologies has further elevated the resolution of promoter screening to the single-cell level. Combined analysis of single-cell RNA sequencing (scRNA-seq) and single-cell ATAC sequencing (scATAC-seq) can simultaneously capture both the transcriptomic expression profile and chromatin accessibility information at the single-cell level, enabling fine-grained dissection of heterogeneous radiation response across cell subpopulations [98]. Since transcriptional responses to equivalent radiation doses differ significantly across cell subpopulations, single-cell multi-omics methods can identify radiation-responsive promoters active only in specific cell subsets (e.g., hematopoietic stem cells, vascular endothelial cells). Gene products regulated by these subpopulation-specific promoters may enter the systemic circulation via exosome secretion or other extracellular signaling pathways, providing potential circulating biomarker targets for non-invasive radiation exposure detection, thereby establishing an important molecular link between intracellular promoter activity and peripheral blood assays [98].

The co-application of computational biology tools is an indispensable component of functional genomics screening strategies. The JASPAR database (the 2020 version contains TF binding matrices from approximately 1800 species) provides rich reference resources for systematic predictive analysis of regulatory elements in candidate promoter regions [99]. In recent years, deep learning models based on convolutional neural networks and Transformer architectures have demonstrated significant potential for promoter activity prediction; among these, the Enformer model can integrate long-range genomic sequence information to directly predict gene expression levels under different conditions, and may play an important auxiliary role in functional prediction and candidate element screening for radiation-responsive promoters [100].

### 4.3. Screening Based on Epigenetic Modifications

Epigenetic modifications—particularly DNA methylation and post-translational histone modifications at promoter regions—constitute key molecular mechanisms governing transcriptional activation and silencing states, while also serving as an important dimension of radiation-responsive regulatory research. Multiple studies have demonstrated that ionizing radiation can induce significant epigenetic remodeling at promoter regions of multiple genes, and these dynamic changes are closely associated with DNA damage repair efficiency, cell cycle checkpoint activation, and cell fate determination [101].

Bisulfite sequencing (BS-seq) is currently the most direct and accurate technical approach for detecting genomic DNA methylation status. Its working principle exploits bisulfite treatment to convert unmethylated cytosines to uracil while leaving 5-methylcytosine unchanged, thereby distinguishing methylated from unmethylated sites at single-base resolution [102]. Using this technology, systematic comparison of genome-wide methylation profiles across different cell lines before and after radiation treatment has revealed that promoter regions of some tumor suppressor genes exhibit demethylation trends following radiation, suggesting that radiation can reactivate transcription of originally silenced genes by altering the epigenetic landscape [101]. ChIP-seq analyses have additionally demonstrated that radiation modulates H3K4me3 (trimethylation of lysine 4 of histone H3, an active transcription mark) at promoter regions of certain DNA repair-related genes, while simultaneously inducing changes in H3K27me3 (trimethylation of lysine 27 of histone H3, a transcriptional repression mark) levels at specific gene promoters, indicating that the radiation response involves coordinated remodeling of chromatin states across multiple layers [103].

O6-methylguanine-DNA methyltransferase (MGMT) gene promoter methylation constitutes the classic example of a deep intersection between epigenetic modification and clinical radiation oncology (discussed in detail in Section 5.3). Hegi et al. demonstrated in a landmark glioblastoma patient cohort study that CpG island methylation in the MGMT promoter leads to transcriptional silencing of this DNA repair enzyme, thereby influencing patient response to combined temozolomide chemotherapy plus radiotherapy and long-term prognosis [41]. This landmark finding not only established MGMT promoter methylation as a core clinical predictive biomarker in glioblastoma but also provided proof-of-concept for the systematic development of epigenetic biomarkers in radiation-related diseases.

However, the limitations of epigenetic modification screening methods warrant equal attention. DNA methylation status is subject to interference from multiple non-radiation factors, including age, tissue type, inflammatory state, and other environmental exposures; single-time-point methylation detection cannot reliably distinguish radiation-specific changes from background biological variation. Furthermore, the high heterogeneity of epigenetic landscapes across different cell types poses challenges for cross-tissue interpretation [41,101]. To overcome these technical limitations, future research should prioritize the development of dynamic analytical frameworks integrating single-cell epigenomics with machine learning algorithms, enabling spatiotemporally specific, precise dissection of radiation-induced promoter epigenetic modifications.

### 4.4. Synthetic Biology and Promoter Engineering Strategies

Synthetic biology employs systematic engineering design principles for directed modification and de novo creation of promoter sequences, providing powerful technical support for functional optimization of radiation-responsive promoters and construction of novel detection systems. An important conceptual boundary requires clarification here: artificially engineered promoters constructed through synthetic biology approaches do not exist in the endogenous human genome and therefore cannot be directly used as biomarkers of radiation exposure in vivo (Track A). Their core application value is manifested at three levels: (i) functional validation, systematically constructing synthetic promoter libraries containing specific regulatory element variants to verify the functional mechanisms of natural responsive elements such as CArG boxes and hypoxia response elements (HREs); (ii) tool development, constructing high-sensitivity in vitro or in vivo reporter gene detection systems (Track B) for radiation dose-monitoring sensor development; and (iii) translational application, providing precisely controllable transcriptional regulatory tools for radiation-induced targeted gene therapy.

In high-throughput screening strategies, massively parallel reporter assay (MPRA) is one of the most rapidly developing synthetic biology screening technologies in recent years. This technology couples synthetic promoter libraries containing different regulatory sequence variants to uniquely barcoded reporter genes, then uses deep sequencing to simultaneously quantitatively evaluate the transcriptional activity of tens of thousands to millions of candidate sequences under radiation conditions, achieving large-scale, precisely quantitative promoter function screening [104]. In combination with systematic evolution of ligands by exponential enrichment (SELEX), researchers can also efficiently screen regulatory elements with optimal response characteristics for specific radiation doses or types from randomized sequence libraries [105]; the combination of these two technologies is expected to substantially accelerate the discovery and functional validation of radiation-responsive elements.

In rational design strategies, the engineering modification of the EGR1 (early growth response protein 1) gene promoter is a classical research example. The EGR1 promoter contains multiple CArG box (consensus sequence CC(A/T)6GG) elements, which are both binding sites for serum response factor (SRF) and key functional sequences mediating radiation-induced transcriptional activation. Hallahan et al. recombined multi-copy CArG box arrays with a minimal core promoter, significantly enhancing the sensitivity and targeting specificity of the artificial promoter to ionizing radiation, and successfully employed this system to drive highly specific expression of therapeutic genes such as tumor necrosis factor in the radiation target area, demonstrating in preclinical models the important translational potential of EGR1 promoter engineering in radiation gene therapy [106].

The introduction of deep learning algorithms is accelerating the design optimization process of synthetic promoters. Convolutional neural networks and Transformer models trained on large-scale promoter sequence-activity datasets can learn complex nonlinear mappings from sequence features to transcriptional activity, enabling efficient exploration of large sequence design spaces at the computational level and substantially shortening the design-build-test-learn (DBTL) engineering iteration cycle [100,107]. This computationally driven design paradigm, combined with high-throughput experimental technologies such as MPRA, is expected to significantly improve design precision and research efficiency in radiation-responsive promoter engineering.

Additionally, programmable transcriptional regulatory systems constructed using CRISPR/dCas9 (catalytically inactive Cas9) provide new research dimensions for dynamic gene regulation in radiation responses. This system can achieve on-demand precise regulation of specific target gene promoters by fusing dCas9 with transcriptional activation or repression effector domains, temporally and spatially mimicking endogenous transcriptional responses induced by radiation [108]. Systematic methodologies accumulated in the field of industrial microbial chassis engineering—including modular regulatory element integration, coordinated promoter and 5′-UTR optimization, and dynamic response system construction—while not directly targeting radiation monitoring, provide valuable technical reference and methodological insights for constructing novel radiation-responsive biosensors [109,110].

## 5. Validation and Applications of Radiation-Responsive Promoters

The research value of radiation-responsive promoters is ultimately realized in their practical translational applications in reporter gene systems, biosensors, dose monitoring, and clinical diagnosis and therapy. Beginning from the construction of functional validation systems, this section systematically describes the research progress in radiation-responsive promoter applications across biosensor design, dose assessment and treatment response prediction, and radiation-guided gene therapy and radiosensitization. Emphasis is placed on distinguishing Track A (biomarker-oriented) from Track B (tool-oriented reporter gene systems) application pathways, with analysis of key technical challenges and future directions in each domain.

### 5.1. Construction and Validation of Reporter Gene Systems

Radiation-responsive reporter gene systems bridge promoter functional research and practical application development, forming the foundation of Track B tool-oriented approaches. A typical system consists of three functional modules in series: radiation-responsive cis-regulatory elements (e.g., CArG boxes or hypoxia response elements HREs), a minimal core promoter sequence (providing the basal transcription initiation platform), and a quantitatively detectable reporter gene (including GFP, luciferase, and sodium-iodide symporter NIS) [106]. The CArG box sequence (consensus CC(A/T)6GG) recruits SRF and mediates potent transcriptional activation of the EGR1 gene under ionizing radiation stimulation, while the HRE element can respond to HIF-1α transcription factor in radiation-induced tumor hypoxic microenvironments; synergistic integration of both significantly broadens the tumor-targeting response range of the system [106,111].

Functional validation of reporter gene systems typically follows a tiered validation strategy proceeding from in vitro to in vivo. The in vitro validation phase employs standardized cellular irradiation experiments using X-rays or γ-rays at different dose rates to systematically quantify the dose-response relationship between reporter gene signal intensity and radiation dose, evaluating system detection sensitivity and linear dynamic range through dose-response curve fitting. The in vivo phase employs subcutaneous or orthotopic tumor models. Bioluminescence imaging (BLI) or positron emission tomography (PET) enables non-invasive, spatiotemporally resolved tracking of reporter gene expression, thereby verifying system functional fidelity in complex tissue microenvironments [111].

Integration of multi-modal molecular regulation strategies has further improved the efficacy of reporter gene systems against radiation-resistant tumors. Studies have demonstrated that KAT5 (lysine acetyltransferase 5) promotes tumor radioresistance through epigenetic regulation via the miR-210/TET2 signaling axis; targeted inhibition of this pathway in combination with radiation-inducible promoter-driven therapeutic gene expression can effectively overcome the radioresistant phenotype, providing new strategies for radiosensitization of refractory tumors [112].

### 5.2. Applications in Radiation Biosensors

Applications of radiation-responsive promoters in biosensors can be categorized by host biological system into prokaryotic (bacterial) systems and eukaryotic/mammalian systems, each with distinctive technical advantages and application positioning.

Prokaryotic bacterial sensor systems are well suited to environmental radiation monitoring owing to their structural simplicity, rapid response, and operational convenience. The recA::luxCDABE system, based on the Escherichia coli SOS response pathway, is the most thoroughly characterized prototype in this field: the recA promoter activates the SOS response upon DNA damage, driving the luciferase operon (luxCDABE) to produce quantitatively detectable bioluminescent signals, with a detection limit as low as 1.5 Gy of γ-ray irradiation [113]. The ColD promoter system uses the SOS regulatory elements of the colicin D operon to drive luciferase reporter gene expression, exhibiting good dose-signal linearity over 0.5–10 Gy, suitable for quantitative detection in the low-to-medium dose range [113].

Eukaryotic system sensors demonstrate technical advantages that more closely reflect human physiological reality in cellular-level radiation dose monitoring. The yeast HUG1p-GFP system uses the promoter of the Saccharomyces cerevisiae DNA damage checkpoint gene HUG1 to drive GFP reporter gene expression, achieving specific fluorescence detection of ionizing radiation-induced DNA damage [114]. In mammalian systems, the CDKN1A-GFP reporter detects DNA double-strand breaks via the p53 pathway, activating the p21 promoter to generate a fluorescence signal that closely mirrors the endogenous human cellular response to radiation [115]. In normal human fibroblasts, CDKN1A expression has been confirmed to correlate significantly with irradiation dose and radiation LET, providing the quantitative basis for a biological dose marker [116]; p21 promoter-based fluorescent reporter cell lines exhibit good dose-dependent fluorescence responses over 2–10 Gy [117], and can be further developed into cell-based sensors supporting real-time quantitative detection at the single-cell level [118]. These studies collectively demonstrate that eukaryotic cell reporter systems, by integrating endogenous damage response pathways, achieve a unification of physiological relevance and detection practicality in cellular-level radiation dose monitoring.

Different radiation biosensor systems exhibit significant differences in performance parameters, primarily in detection limits (prokaryotic systems ~0.5–2 Gy; eukaryotic systems as low as 0.1 Gy), linear dynamic range (typically 1–2 orders of magnitude), response time from radiation exposure to peak signal (bacterial systems 1–4 h; mammalian cell systems 4–24 h), and cross-reactivity with non-radiation factors such as chemical toxicity and oxidative stress [113,119]. Comprehensive optimization of system performance requires seeking a reasonable balance among high sensitivity, broad linear range, and high response specificity.

### 5.3. Applications in Radiation Dose Assessment, Monitoring, and Treatment Response Prediction

#### 5.3.1. Real-Time Radiation Dose Monitoring

Real-time radiation dose monitoring is a critical requirement in radiation protection and radiotherapy quality control; promoter-driven reporter gene systems offer promising molecular solutions. Real-time monitoring systems driven by the CDKN1A (p21) promoter have demonstrated several important functional characteristics, including non-linear dose-dependent induction kinetics under continuous low-dose radiation and reinducibility, defined as the maintenance of stable responses across successive radiation–recovery–re-irradiation cycles, suggesting potential capability for adapting to chronic radiation exposure scenarios [117].

The third-generation promoter probe vector pKG represents an important advance in radiation-responsive reporter system design. Using the radiation-inducible responsive motif (RDRM) as the core regulatory element, this vector achieves near-zero basal expression through precision promoter engineering, elevating the radiation-induced signal-to-noise ratio to several times that of first- and second-generation systems; over the range of 0.5–8 Gy, GFP fluorescence signal intensity correlates positively with radiation dose, with potential for quantitative detection in the low-dose range [119].

Nevertheless, promoter-based real-time in vivo dose monitoring technology currently faces several critical bottlenecks: non-invasive penetrating readout of in vivo reporter gene signals (particularly the attenuation of GFP fluorescence signals in deep tissue) substantially limits in vivo applicability; quantitative conversion of dynamic reporter gene signal decay during cellular damage repair to radiation dose remains without standardized models; and radiation sensitivity differences across different tissue cell types may cause significant fluctuations in reporter signal intensity at the same dose, increasing the complexity of cross-tissue calibration [119]. Addressing these issues requires synergistic advances in delivery system engineering and multimodal signal readout technologies.

#### 5.3.2. Treatment Response Prediction: Promoter Methylation Markers

Promoter methylation status, as an epigenetic biomarker, has demonstrated important clinical value in predicting tumor responses to chemoradiotherapy, a core Track A (biomarker-oriented) application.

MGMT (O6-methylguanine-DNA methyltransferase) gene promoter methylation is the most thoroughly investigated and clinically best-evidenced predictive epigenetic biomarker for tumor chemoradiotherapy to date. Its regulatory mechanism is as follows: CpG island hypermethylation in the MGMT promoter region leads to transcriptional silencing of this DNA repair enzyme, causing tumor cells to lose the capacity to remove O6-alkylguanine adducts induced by temozolomide (TMZ), thereby leading to persistent DNA damage accumulation and ultimately apoptosis sensitization [41]. In a landmark randomized controlled study of 206 glioblastoma patients, Hegi et al. confirmed that the median overall survival of patients with MGMT promoter methylation receiving TMZ combined with radiotherapy reached 21.7 months, significantly superior to 12.7 months in unmethylated patients (HR = 0.51, *p* < 0.001), establishing MGMT promoter methylation testing as the gold standard for treatment decision-making in glioblastoma [41]. Advances in liquid biopsy technology have further expanded the clinical accessibility of MGMT methylation testing: the concordance between tumor tissue and matched plasma circulating tumor DNA (ctDNA) MGMT methylation status has been reported to reach 87.5% (Cohen’s Kappa = 0.75), providing an important technical foundation for non-invasive dynamic monitoring during treatment and early warning of disease progression [120].

PAX1 (paired box transcription factor 1) gene promoter methylation has demonstrated notable clinical value in predicting chemoradiotherapy responses in cervical cancer. Quantitative analysis of PAX1 promoter methylation in cervical cancer patients by quantitative methylation-specific PCR (qMSP) revealed significant differences in methylation levels between complete and non-complete treatment responders (ΔCp values: complete response group 5.08 vs. non-complete response group 17.35, *p* < 0.05), suggesting that PAX1 promoter hypermethylation may serve as a potential molecular indicator for predicting complete response to concurrent chemoradiotherapy, with application prospects for guiding individualized treatment decisions [121].

TERT (telomerase reverse transcriptase) promoter methylation reflects important diagnostic value in predicting the invasiveness of pituitary neuroendocrine tumors. Studies have found significant positive correlations between TERT promoter methylation status and the invasive phenotype, post-surgical recurrence risk, and Ki-67 proliferation index of pituitary tumors, potentially serving as an ancillary diagnostic marker for distinguishing indolent from aggressive pituitary neuroendocrine tumors, providing a reference for patient stratification management and radiotherapy indication selection [122].

#### 5.3.3. Genetic Polymorphisms and Radiation Sensitivity

Genetic polymorphisms in promoter regions modulate inter-individual differences in radiation response by altering transcription factor (TF) binding efficiency and downstream gene expression, thereby providing an important genetic basis for precision radiation protection and individualized radiotherapy planning.

Genetic polymorphisms in the VIM-AS1 lncRNA gene promoter region are closely associated with individual susceptibility to radiation-induced fibrosis. Studies have found that VIM-AS1 promoter polymorphism sites can regulate VIM-AS1 expression levels, subsequently influencing the activity of the TGFB1/VIM (transforming growth factor β1/vimentin) signaling axis, ultimately modulating the histological progression and severity of radiation-induced fibrosis [88]. This finding reveals a new mechanism by which lncRNA regulatory networks participate in the genetic determination of late radiation side effects, providing a potential genetic biomarker for predicting post-radiotherapy fibrosis risk.

The ITGAM (integrin αM subunit) gene promoter region −323G>A SNP shows a significant statistical association with nutritional injury risk following radiotherapy for head and neck tumors. Functional studies indicate that the −323G>A variant alters GABP (GA-binding protein) TF binding efficiency at this site, influencing the basal transcriptional activity of the ITGAM gene and thereby regulating integrin αMβ2-mediated immune cell inflammatory functions; patients carrying the A allele exhibit an approximately 14-fold reduction in risk of severe nutritional injury complications following intensity-modulated radiation therapy for head and neck cancer compared to G allele carriers (OR = 0.07, 95% CI: 0.01–0.62), suggesting that ITGAM promoter polymorphism has potential clinical value as a predictor of radiotherapy toxicity [123,124]. Together, these studies outline the core molecular logic chain by which promoter polymorphisms regulate inter-individual differences in radiation effects: genetic polymorphism → altered promoter regulatory element activity → protein expression level differences → individual variation in radiation-related biological effects, providing an important research paradigm for the systematic discovery of future radiation sensitivity genetic markers.

#### 5.3.4. Future Diagnostic Application Directions

With the continued maturation of multi-omics technology frameworks and computational biology tools, promoter biology-based radiation diagnostic applications are accelerating toward multi-dimensional integration and precision personalization. Construction of multi-modal promoter sensor arrays represents one of the most promising technical development directions in this field: by integrating promoters responsive to different radiation dose thresholds with multi-color fluorescent reporter genes or orthogonal signaling elements in parallel, multiple biological signals including DNA damage responses, oxidative stress, and epigenetic remodeling can be simultaneously captured, achieving more precise integrated radiation dose and biological effect assessment compared to single markers [125].

At the computational diagnostic model level, multi-dimensional integration of promoter methylation status profiles, TF binding profiles (from ChIP-seq data), and chromatin accessibility (from ATAC-seq data) can establish multi-layer regulatory feature models with more comprehensive predictive efficacy than single methylation detection, potentially achieving higher sensitivity and specificity in tumor chemoradiotherapy response prediction [125,126]. Deep integration of liquid biopsy and ctDNA technology provides a non-invasive detection pathway for the above diagnostic strategies: longitudinal dynamic monitoring of ctDNA promoter methylation status in peripheral blood enables real-time tracking of the molecular response process of tumors to radiotherapy, enabling early prediction of therapeutic efficacy and timely intervention decisions [127]. This integrated strategy merging molecular diagnostics with precision therapeutic monitoring heralds an increasingly important role for radiation-responsive promoter biology in the era of liquid biopsy-driven precision oncology.

### 5.4. Applications in Radiation-Guided Gene Therapy and Radiosensitization

#### 5.4.1. Radiation-Inducible Therapeutic Gene Expression

Radiation-inducible therapeutic gene expression systems utilize radiation-responsive promoters to precisely couple local tumor radiotherapy with targeted therapeutic gene activation in both spatial and temporal dimensions, fundamentally overcoming the insufficient tumor targeting of traditional systemic drug delivery approaches. This represents the most clinically translatable research direction within Track B applications. The EGR1 gene promoter—harboring 5 functional CArG box (CC(A/T)6GG) elements in its promoter region—has become the most widely studied radiation-responsive driving element in this field: under 2–10 Gy X-ray irradiation, EGR1 promoter activity can be upregulated 4–10-fold, sufficient to drive high-level specific expression of downstream therapeutic genes in the radiation target area [106,111].

The combined radiation-gene therapy strategy employing EGR1 promoter-driven TNF-α expression has undergone systematic preclinical and early clinical evaluation. Hallahan et al.’s original research demonstrated that high-level TNF-α expression in tumor tissue triggered by local tumor irradiation (5 Gy), activating the EGR1 promoter, can produce significant synergistic killing effects with radiotherapy; the Phase I clinical trial (TNFerade study) also preliminarily validated the safety and biological activity of this strategy in solid tumor patients [106]. Additionally, γ-ray irradiation can selectively enhance hTERT (human telomerase reverse transcriptase) promoter transcriptional activity in laryngeal squamous cell carcinoma, providing new theoretical foundations for constructing composite targeted therapy systems that use TERT promoters to drive radiation-sensitizing suicide genes [128].

#### 5.4.2. Multi-Modal Responsive Promoter Systems

Single radiation stimulus-driven therapeutic gene systems have an inherent limitation of low response efficiency in hypoxic tumor regions (typically radiotherapy-resistant zones); radiation-hypoxia dual-responsive chimeric promoter systems were developed to compensate for this deficiency. By modular tandem integration of CArG elements from the EGR1 promoter with HIF-1α-responsive HRE (hypoxia response element) sequences, the constructed chimeric promoter can individually or synergistically activate downstream therapeutic gene expression under both radiation and tumor hypoxia signal conditions, effectively expanding the tumor-targeted coverage of therapeutic gene activation, particularly suitable for targeted intervention in hypoxic radioresistant subregions of solid tumors [129].

The 131I internal irradiation-driven EGR1/CArG positive feedback amplification system achieves innovative functional integration of radionuclide therapy with gene therapy: β-ray irradiation from 131I activates CArG box-mediated EGR1 promoter, driving upregulation of sodium-iodide symporter (NIS) gene expression; increased NIS protein expression leads to enhanced tumor cell iodine uptake capacity; and uptake of more 131I produces stronger radiation signals, forming a positive feedback amplification loop that realizes radiation-triggered self-amplifying therapeutic effects [130,131]. The heat-inducible protein HSPA6 (Hsp70B) promoter has also been explored for driving targeted therapeutic gene delivery in mesenchymal stem cells (MSCs): mild hyperthermia induces HSP70B promoter activation, triggering therapeutic gene expression at tumor sites homed by MSCs within a predetermined time window, achieving temporally controllable tumor-targeted gene delivery [132].

#### 5.4.3. Oncolytic Virus and Radiotherapy Combination Therapy

The tumor-specific transcriptional activity of the TERT promoter provides precise tumor-targeting logic for constructing radiation-virus synergistic oncolytic therapy systems. The hTERT promoter is highly active in 85–90% of malignant tumors while exhibiting extremely low transcriptional activity in normal differentiated cells; this highly tumor-specific transcriptional profile confers unique advantages as a driving element for conditionally replicating adenoviruses (CRAds). Replacing the natural promoter of the adenoviral E1A gene with the hTERT promoter creates tumor-targeted CRAds that can complete replication cycles and mediate cell lysis only in TERT-positive tumor cells; when combined with fractionated radiotherapy, both demonstrate significant synergistic enhancement in tumor DNA damage accumulation and immunogenic cell death [133,134,135,136,137].

#### 5.4.4. Epigenetic Sensitization and Promoter Reactivation

Promoter reactivation strategies mediated by DNA demethylation represent an important mechanism of synergistic sensitization between epigenetic drugs and radiotherapy. Studies have demonstrated that the DNA methyltransferase inhibitor 5-aza-2′-deoxycytidine (5-aza-dC) can reverse CpG hypermethylation at the ATM (ataxia-telangiectasia mutated kinase) gene promoter region, reactivating ATM transcription, restoring its critical regulatory function in DNA DSB repair, and thereby enhancing tumor cell sensitivity to subsequent radiotherapy [138]. This epigenetic pretreatment-radiotherapy sequential strategy reveals a new pathway for achieving individualized radiosensitization at the molecular target level, particularly important for tumor subtypes with relatively low ATM expression that exhibit relative radiotherapy resistance.

#### 5.4.5. Principal Challenges and Outlook

Despite the accumulation of significant encouraging preclinical research evidence for radiation-responsive promoters in gene therapy and radiosensitization, their translational applications continue to face three core challenges. First, spatiotemporal precision optimization: precise matching of the promoter activation time window with radiotherapy fractionation schemes, high spatial coincidence between therapeutic gene expression distribution and the tumor target region, and avoidance of unintended bystander effects in adjacent normal tissues represent critical technical bottlenecks constraining clinical translation efficiency. Second, delivery system improvement: development and optimization of highly efficient tumor-targeted gene delivery vehicles (including adeno-associated virus AAV, lipid nanoparticles LNP, and engineered exosomes) and overcoming tumor microenvironment barriers to achieve sufficient transfection are pressing engineering challenges. Third, clinical translation standardization: establishing unified in vivo reporter gene expression monitoring standards, standardized dose-effect correlation evaluation systems, and clinical trial design frameworks meeting regulatory requirements are necessary conditions for translating research achievements in this field into clinical practice. Looking to the future, with continued advances in precision gene delivery technology, AI-driven promoter design optimization, and multimodal real-time imaging, radiation-responsive promoter-mediated gene therapy systems are expected to achieve systematic translation from laboratory to clinic in the not-too-distant future.

## 6. Challenges and Future Directions

The radiation-responsive promoter research field has accumulated substantial basic and translational research achievements over the past two decades; however, a considerable gap remains between laboratory discovery and clinical application. This section, based on a systematic review of current core challenges in the field, provides a forward-looking analysis of future research development directions.

### 6.1. Current Challenges

#### 6.1.1. Insufficient Practical Evidence

An honest assessment of the field reveals a significant evidence gap between promoter-based radiation biomarkers and established clinical standards. In the radiation biodosimetry domain, dicentric chromosome analysis and γ-H2AX foci counting remain the most widely clinically validated gold-standard biodosimetric methods: the former, systematically standardized by IAEA technical documents, enables reliable dose estimation over the range of 0.1–20 Gy; the latter offers high sensitivity (detection limit as low as 10 mGy) and rapid response, with rigorous validation in multiple multi-center prospective studies [139,140]. By contrast, promoter-based biomarkers—including radiation-responsive promoter-driven reporter gene signals and promoter methylation state changes—have not yet undergone the same level of systematic clinical validation as these gold-standard methods; evidence-based medical data on their accuracy, reproducibility, and cross-population applicability remain severely insufficient.

The primary technical bottleneck facing current promoter-based biomarker research is the absence of standardized systematic validation procedures. The overwhelming majority of existing studies have not systematically established complete dose-response curves; LOD and quantitative linear range lack consistent definition across different research reports; and systematic comparison of marker response characteristics across different dose rates, different radiation types (photon versus particle radiation), and different exposure fractionation schemes (acute versus chronic low-dose-rate exposure) has not been conducted [79]. The ICRP has explicitly stated in relevant biodosimetry guidance documents that clinical translation of any candidate biodosimeter must satisfy three core criteria: dose-response linearity, low coefficient of variation (CV < 20%), and cross-laboratory reproducibility, yet existing promoter-based marker studies generally fail to provide systematic performance data on these critical parameters.

Poor cross-study reproducibility represents another core challenge limiting field progress. Cell line models used in most studies (typically highly passaged tumor cell lines) differ fundamentally from primary human cells in radiation response characteristics, and species-level differences in radiation sensitivity and promoter regulatory networks between animal models (primarily mice) and humans further amplify uncertainty in result extrapolation [27]. Most critically, many studies remain at the in vitro or mouse model stage, with clinical-grade evidence essentially absent; no promoter-based biomarker assay has yet completed multi-center prospective clinical validation meeting international standards, representing a fundamental barrier to clinical translation [79]. Systematically addressing this evidence deficit is the primary prerequisite for advancing radiation-responsive promoter research from basic exploration to practical application.

#### 6.1.2. Data Standardization and Sharing

Absence of data standardization and sharing represents a systemic obstacle constraining overall progress in the radiation-responsive promoter research field. At the detection platform level, different research institutions employ various technical approaches for promoter methylation analysis—including pyrosequencing, methylation-specific PCR (MSP), bisulfite sequencing (BS-seq), and multiplex ligation-dependent probe amplification (MLPA)—with significant differences in detection sensitivity, coverage, and quantitative precision across platforms [141]. At the data analysis pipeline level, differences in bioinformatics pipelines (including alignment software versions, methylation calling algorithm parameters, and differential analysis statistical models) can cause fundamentally different biological conclusions from identical raw sequencing data. Even within studies employing the same WHO CNS classification standards for sample selection, different laboratories integrating genomic and transcriptomic data may obtain substantially divergent prognostic stratification results, severely limiting reproducibility and cross-center comparability [142].

At the data sharing level, the radiation biology field currently lacks standardized data storage and exchange format specifications specifically for promoter-based radiation response markers. Although general genomic databases such as NCBI GEO and ArrayExpress provide basic platforms for open data sharing, the absence of structured metadata standards containing detailed radiation exposure parameters (dose, dose rate, radiation quality) and biological background information (tissue type, species, treatment time window) means that integrative cross-institutional and cross-species dataset analyses face serious information gaps. Establishing unified standard operating procedures (SOPs) and FAIR-compliant (Findable, Accessible, Interoperable, Reusable) data sharing mechanisms represents a foundational infrastructure challenge requiring urgent resolution in this field [143].

#### 6.1.3. Technical Bottlenecks in Multi-Omics Integrative Analysis

The inherent limitations of single-omics data in dissecting radiation response mechanisms have driven the development of multi-omics integration research strategies; however, multi-omics integrative analysis currently faces several unresolved core bottlenecks at the technical implementation level.

From a data integration methodology perspective, algorithmic frameworks capable of effectively handling spatiotemporal heterogeneity and high-dimensional nonlinear associations in multi-omics data remain lacking. Genomic variants (e.g., IDH1/2 mutations, TERT promoter mutations), epigenomic modifications (e.g., promoter CpG methylation, H3K27me3 distribution), transcriptomic features (mRNA and non-coding RNA expression profiles), and proteomic data each have different data dimensions, noise structures, and missing value patterns; conventional linear dimensionality reduction and regression analysis methods cannot adequately capture complex interactions across different omics layers [142,144]. Although methods such as factor analysis (MOFA+), multi-kernel learning, and graph neural networks have shown some promise in multi-omics integration, their biological interpretability and cross-dataset generalizability still require systematic evaluation [144].

At the single-cell resolution level, although combined analysis of scRNA-seq and scATAC-seq can simultaneously resolve transcriptomic states and chromatin accessibility at single-cell resolution, radiation-induced cell population heterogeneous responses—including the dynamic emergence of subpopulations with different radiation sensitivities and real-time remodeling of their promoter regulatory networks—remain technically difficult to track in high-throughput time-series [98]. Furthermore, the inherent sparsity of single-cell epigenomics data (limited genomic sites covered per cell) introduces substantial statistical uncertainty in methylation state inference at promoter regions, particularly at low radiation doses, where radiation-specific epigenetic signals may be masked by stochastic intercellular variation [98]. The introduction of AI and machine learning provides important tools for constructing promoter activity prediction models, but model interpretability, coverage of radiation-exposure-specific training data, and generalizability in independent validation cohorts remain key uncertainties constraining practical application [100].

#### 6.1.4. Clinical Translation Bottlenecks

Clinical translation bottlenecks are primarily manifested across three interconnected dimensions: insufficient mechanistic elucidation, absence of detection technology standardization, and ambiguous positioning of clinical application scenarios. At the mechanistic level, although promoter methylation is known to influence radiosensitivity by regulating tumor suppressor genes (e.g., integrin β1) or key signaling pathways (e.g., PI3K/AKT), the causal relationships, temporal sequence dependencies, and cooperative regulatory mechanisms with DNA damage repair pathways between radiation-induced promoter methylation dynamic changes and specific signal transduction pathways remain without systematic mechanistic analysis [41].

At the detection technology level, most existing promoter-based biomarker assays still rely on tissue biopsy samples, creating a fundamental operational feasibility conflict with the clinical requirement for dynamic monitoring during radiotherapy. Liquid biopsy technology (including circulating tumor DNA methylation detection and circulating miRNA and small RNA profiling) provides important technical pathways for overcoming this limitation; however, the sensitivity and specificity of current liquid biopsy methods in radiation-specific biomarker detection still require optimization: ctDNA abundance in plasma is extremely low (typically <0.01% in healthy individuals), and its magnitude of change following low-dose radiation exposure may fall below the detection threshold of existing sequencing methods [145]. At the application scenario level, positioning of promoter-based markers (acute high-dose exposure assessment, chronic low-dose occupational monitoring, or tumor chemoradiotherapy response prediction) has not yet formed a systematic research framework precisely matched to corresponding clinical needs, directly causing non-targeted research design, inconsistent validation standards, and consequently slowing the production of clinical-grade evidence [79].

### 6.2. Future Research Directions

#### 6.2.1. Interdisciplinary Collaboration and Technology Fusion

Driving systematic breakthroughs in the radiation-responsive promoter research field requires deep technology fusion at multiple disciplinary intersections, constructing a problem-oriented interdisciplinary collaborative research system.

Deep interdisciplinary fusion of epigenetics and radiation biology is the core pathway for revealing the molecular mechanisms by which promoters regulate radiation responses. By systematically dissecting the quantitative coupling relationships between radiation-induced DNA methylation dynamic changes (encompassing time-dependent, dose-dependent, and cell-type-specific dimensions) and DNA DSB repair efficiency and cell cycle checkpoint activation, causal molecular models connecting epigenetic remodeling with cellular radiation response phenotypes can be established, providing a solid mechanistic foundation for developing early radiation exposure biomarkers based on promoter methylation changes [41,101].

AI-driven multi-modal data integration models represent one of the most transformative technical development pathways in this field. Integrating ChIP-seq, ATAC-seq, and Hi-C (chromatin conformation capture) multi-dimensional genomic data as input for deep learning models enables high-precision prediction of promoter activity dynamic changes post-radiation while accounting for long-range regulatory interactions and three-dimensional genomic structure; constructing promoter-enhancer regulatory networks using graph neural networks (GNNs) can systematically reveal remodeling patterns of transcriptional regulatory logic circuits under radiation conditions, providing computationally driven discovery pathways for identifying novel core radiation-responsive regulatory nodes [100,146]. Simultaneously, advances in nanotechnology and gene delivery provide new solutions to the delivery efficiency bottleneck in clinical translation of radiation-responsive promoter-driven therapeutic gene systems: next-generation delivery vehicles such as targeted lipid nanoparticles (LNPs) and engineered exosomes may enable highly efficient targeted delivery of radiation-responsive promoter-therapeutic gene complexes to tumor sites, further improving the therapeutic window ratio of these systems [147].

#### 6.2.2. Standardized Database and Tool Development

Building standardized databases specific to radiation-responsive promoters represents an important infrastructure engineering project supporting the systematic development of this field. Based on data hierarchy and application requirements, the database architecture can be designed as a three-tier progressive system: the first tier constitutes a clinical sample resource library containing prospective cohort sample information with complete radiation exposure parameter records (dose, dose rate, radiation type, fractionation scheme) and longitudinal follow-up data; the second tier constitutes a molecular detection raw data repository encompassing whole-genome sequencing, bisulfite methylome, single-cell transcriptome, and multi-dimensional chromatin state data, with standardized bioinformatics analysis pipelines; the third tier constitutes a phenotype-genotype association knowledge graph providing a searchable, updateable knowledge platform through integration of multi-dimensional association networks linking promoter variants, methylation status, and radiation biological effect endpoints (DNA damage repair kinetics, cell survival curve parameters, clinical toxicity scores).

At the diagnostic tool development level, priority should be placed on advancing two categories of key technology products: portable promoter mutation and methylation rapid detection devices that integrate CRISPR-Cas12a/Cas13-mediated specific nucleic acid recognition with lateral flow immunochromatographic strips, enabling point-of-care minute-scale quantitative detection and extending promoter-based biomarker testing from specialized laboratories to frontline clinical and field radiation accident sites, and cloud computing platform-supported real-time multi-omics analysis pipelines that use standardized containerized computing environments (e.g., reproducible analysis workflows based on Docker/Singularity) to achieve cross-institutional, cross-platform standardized integration analysis of multi-omics data while constructing machine learning inference models for radiation exposure dose estimation and promoting the formation of standardized biodosimetry protocols [143,148].

#### 6.2.3. Clinical Application Scenario Expansion

Clinical application expansion of radiation-responsive promoter biology can proceed systematically along three dimensions with inherent logical connections. The first dimension involves systematically incorporating promoter biomarkers (particularly promoter mutations and methylation status of TERT, IDH1/2, and other genes) into existing tumor molecular classification systems as supplementary molecular indicators to current diagnostic frameworks such as the WHO CNS5 classification, to enhance the predictive efficacy of classification systems for chemoradiotherapy responses and prognosis assessment; it is important to note that existing evidence indicates that simply adding transcriptomic feature analysis to current molecular classification systems does not necessarily improve prognostic stratification efficacy, and future research should focus on dissecting the functional consequences of promoter variants rather than remaining at the statistical association level [41,142].

The second dimension involves developing companion diagnostic products with clinical application value. Plasma cfDNA-based promoter methylation quantification assay kits and serum-based small RNA (including tsRNA and miRNA) multiplex detection panels can serve as early molecular response indicators and adverse event prediction tools for radiotherapy efficacy, enabling full-course dynamic molecular monitoring of the radiotherapy process and supporting adaptive radiotherapy dose adjustment decisions based on molecular response information [145,149]. Collaborative exploration with tumor immunotherapy represents another important translational research opportunity: TERT promoter mutations may alter tumor antigen presentation and immune editing processes to change the immune infiltration state of the tumor microenvironment, thereby influencing PD-1/PD-L1 inhibitor treatment responses; systematic validation of this hypothesis is expected to provide molecular decision-making evidence for radiation-immunotherapy combination strategies [150].

The third dimension involves extending the application scenario from oncology to occupational radiation health monitoring and environmental radiation contamination assessment. For chronically low-dose-irradiated occupational groups such as nuclear power plant workers and interventional radiologists, establishing cumulative exposure assessment systems based on dynamic monitoring of promoter methylation profiles in peripheral blood nucleated cells can serve as an important biological supplementary verification tool for existing physical dosimeters. For environmental radiation contamination scenarios such as nuclear accidents, developing mobile biological dose assessment systems integrating rapid blood sampling with immediate detection of promoter-based markers has the potential to elevate population triage efficiency from the current hundreds per day to the thousands-per-day level, providing critical technical support for medical emergency responses to large-scale nuclear and radiation incidents [79,139].

Taken together, the radiation-responsive promoter research field stands at a critical historical juncture, transitioning from basic mechanistic exploration to translational research. Only by honestly confronting the deep-seated issues of insufficient practical evidence, absent data standardization, and unclear clinical translation pathways—systematically completing the evidence chain through multi-center prospective clinical validation studies, breaching multi-omics integrative analysis bottlenecks through interdisciplinary technology fusion, and consolidating the foundation for data sharing and tool development through infrastructure investment—can this field advance from promising scientific discovery to a practical technology system with genuine clinical value.

## 7. Conclusions

This review systematically analyzed radiation-responsive promoters with respect to their molecular types and regulatory mechanisms, screening strategies, functional validation, translational applications, and future challenges, providing an integrative reference for advancing research and clinical translation in this field.

At the level of promoter molecular types and mechanisms, five major classes of radiation-responsive transcriptional regulatory units were systematically summarized. miRNA promoters (represented by miR-21 and miR-34a) can achieve transcriptional activation through the ATM/p53 signaling axis at radiation doses as low as 0.5 Gy, demonstrating high dose sensitivity [151]; tsRNA promoters, as recently identified novel radiation-responsive regulatory elements, have radiation specificity closely associated with the transcriptional regulatory characteristics of their parent tRNA genes [152]; Acute-phase protein gene promoters (e.g., CRP, SAA1) mediate radiation-induced transcriptional activation of systemic inflammatory responses through NF-κB and STAT3 signaling [153]. DNA damage repair gene promoters (represented by CDKN1A/p21 and GADD45A) exhibit dose-dependent transcriptional upregulation mediated through p53 response elements and DDREs [79]. lncRNA promoters participate in regulating genomic stability following radiation stress through chromatin remodeling and competitive transcription factor binding. These five categories of regulatory units are driven by different TF-responsive element axes, including EGR1/CArG boxes, p53 response elements, NF-κB binding sites, HIF-1α/HREs, and ATF/CREB, with effective response dose ranges spanning from the milligray scale (miRNA class) to tens of grays (acute-phase protein class), collectively constituting a multi-tier molecular network of radiation-responsive transcriptional regulation.

At the application positioning level, this review explicitly distinguishes two parallel application tracks for radiation-responsive promoter research to avoid conceptual conflation. Track A (biomarker-oriented): radiation-responsive promoters and their regulated gene products naturally present in the biological genome (including plasma circulating miRNAs, peripheral blood mRNA expression profile changes, and promoter methylation status) serve as molecular evidence for radiation exposure, supporting biological dose assessment of radiation exposure, occupational chronic exposure monitoring, and population triage in nuclear/radiation incidents [154,155]. Track B (tool-oriented): through synthetic biology engineering, integration of radiation-responsive elements, artificial promoter-driven reporter gene detection systems and radiation-inducible therapeutic gene expression vectors are constructed, supporting the development of radiotherapy efficacy monitoring sensors and radiation-guided targeted gene therapy [106,111]. These two tracks have distinct emphases in technical pathways, validation standards, and translational objectives and should be clearly distinguished in research design and clinical evaluation.

In an objective assessment of current research progress, it is necessary to note that despite the valuable basic data accumulated in the above studies, a substantial evidence gap still exists between promoter-based biomarkers and the current clinical gold standards—dicentric chromosome analysis and γ-H2AX foci counting. Dicentric chromosome analysis, standardized by IAEA technical documents, enables reliable retrospective dose estimation over a broad dose range of 0.1–20 Gy; γ-H2AX foci counting has a detection limit as low as 10 mGy and has been rigorously validated in multi-center prospective studies [27,139]. By contrast, current promoter-based marker studies generally lack complete dose-response curves, systematic LOD data, and cross-laboratory reproducibility validation, with clinical-grade multi-center prospective evidence essentially absent [14]. However, promoter-based biomarkers also possess unique potential advantages not shared by cytogenetic methods: plasma-based miRNA/tsRNA detection and ctDNA methylation analysis are technically amenable to completely non-invasive sampling with operational feasibility for real-time dynamic monitoring; promoter methylation status changes (e.g., MGMT promoter methylation) as tumor chemoradiotherapy response prediction markers have received preliminary clinical validation, demonstrating a realistic pathway for extended application to precision tumor therapy [41]. Rational assessment of this evidence status and these unique advantages can guide the allocation of research resources in this field toward the most genuine translational potential.

Based on this comprehensive analysis, this review proposes that future radiation-responsive promoter research should focus on the following four core directions. First, systematically establishing dose-effect quantitative relationships: for each category of candidate markers, systematically determining LOD, linear dynamic range, and uncertainty parameters under conditions covering low doses (<0.1 Gy) to high doses (>10 Gy), acute versus chronic fractionated exposure, and different radiation qualities (photon versus particle radiation), constructing performance datasets meeting international biodosimetry standards [14]. Second, conducting systematic validation with multi-center prospective clinical studies as the core: transcending current limitations of primarily in vitro cell line and animal model research to complete independent validation of candidate markers in real clinical cohorts (encompassing radiotherapy patients, occupationally exposed populations, and radiation accident survivors), which is the indispensable path for translating basic research discoveries into practical diagnostic tools [27]. Third, constructing multi-omics integrative analysis platforms: systematically integrating promoter methylome, transcriptome, proteome, and metabolome data, combined with AI-assisted prediction models, to establish radiation response comprehensive assessment systems more robust than single markers, while advancing database construction adhering to FAIR principles and cross-institutional data sharing standards [143,144]. Fourth, advancing standardized detection tool product development: developing integrated CRISPR nucleic acid detection portable devices for point-of-care testing scenarios and developing high-throughput blood miRNA/methylation rapid detection platforms for large-scale emergency responses to meet the practical needs of rapid population triage at the thousands-of-persons scale in nuclear and radiation incidents [155].

Looking to future application prospects, radiation-responsive promoter biology is expected to play an increasingly significant role in two important domains. In precision radiotherapy, epigenetic biomarkers represented by MGMT and PAX1 promoter methylation status hold the potential to advance radiotherapy protocols from empirical dose prescription based on anatomical imaging to adaptive individualized treatment based on real-time molecular responses, providing molecular decision support for improving local tumor control rates and reducing late adverse effects in normal tissues [41,140]. In nuclear and radiation emergency medical response, biological dosimetry methods centered on gene expression profiles and promoter-based markers can serve as high-throughput supplementary tools to traditional cytogenetic methods, playing a critical role in rapid medical triage following large-scale human irradiation events, together with physical dose reconstruction, clinical symptom assessment, and traditional biodosimeters to constitute a multi-tier, complementary, comprehensive dose assessment system [139,155]. Overall, translating the research potential of this field into tangible clinical and public health benefits requires sustained collaboration among radiation biology, epigenetics, computational biology, and clinical medicine to systematically close the current practical evidence gap through evidence-based scientific approaches, advancing radiation-responsive promoter research into a new stage of evidence-based translational application.

## Figures and Tables

**Table 1:** Systematic comparison of core technical parameters across major radiation biomarker classes.

Parameter	Dicentric Chr. (DIC Assay)	Micronucleus (CBMN Assay)	Gamma-H2AX Foci Count	Promoter-Based Transcription	Key References
Dose range (optimal linear)	0.1–5 Gy	0.2–4 Gy	0.01–10 Gy	Variable; 0.1–10 Gy (gene-specific)	[15,39,40]
Diagnostic time window	48–72 h (lymphocyte culture req.)	72 h (culture required)	Peak 30–60 min; resolution ~24 h (repair-dep.)	Minutes to days; some weeks (gene-specific)	[40,41]
Detection matrix	Peripheral blood lymphocytes (PBLs)	PBLs	PBLs; tissue biopsy	PBLs; plasma (circulating RNA); tissue	[39,40]
Inter-individual variability	Moderate (age, prior exposure)	Moderate	Moderate (repair kinetics vary by individual)	Potentially high (methylation; SNPs; inflammation)	[39,40]
Non-invasive potential	No (venipuncture)	No (venipuncture)	No (venipuncture)	Partial: yes (plasma miRNA; tsRNA)	[40]
Radiation-type selectivity	High-LET > low-LET (excess DIC/dose)	Moderate LET-dependence	LET-dependent foci complexity	Gene-specific; some show quality dependence	[42]
Clinical validation level	IAEA gold std.; ISO standardized	Validated; high-throughput capable	Extensively valid. in vitro/in vivo	Limited; mostly preclinical; except MGMT methylation	[15,40,41]
Multiplexing capability	Low	Low	Moderate	High (transcriptome scale)	–

**Table 2:** Comparative overview of major radiation-responsive promoter classes and their biomarker properties.

Promoter Class	Representative Molecules	Key Activating Transcription Factors	Dose Range (Optimal)	Kinetics (Peak After IR)	Biomarker Detection Matrix	Clinical Evidence Level
miRNA promoter	miR-21, miR-34a, miR-140, miR-7	p53, NRF2, NF-kappaB, AP-1	0.5–10 Gy	6–48 h (varies by miRNA)	Serum/plasma qRT-PCR	Moderate; confounders limit specificity
tsRNA promoter	tRF-Gly-GCC, 15 tsRNA panel	Under active investigation	0.5–4 Gy (carbon ion/proton)	30 min (acute); days (sustained)	Plasma small RNA-seq/qPCR	Preliminary; no clinical standard yet
Acute-phase Protein promoter	SAA, CRP, IL-6, IL-1Ra	NF-kappaB, C/EBPbeta, STAT3	1–10 Gy (systemic)	12–72 h (protein level)	Serum ELISA; plasma protein array	Moderate; poor radiation specificity
DNA repair Gene promoter	CDKN1A(p21), GADD45A, MGMT, BEND4	p53, AP-1, NF-kappaB, NRF2	0.1–10 Gy (p21: linear 0.1–2 Gy)	2–12 h mRNA; 6–24 h protein	PBL RT-qPCR; promoter methylation PCR	Good (p21/GADD45); excellent (MGMT methylation)
lncRNA promoter	LINP1, ZFAS1, VIM-AS1, HAR lncRNAs	Cell-type specific; multiple TFs	Tissue-specific; range unclear	Hours to days	Tissue biopsy; poor plasma detection	Preliminary; highly tissue-specific

## Data Availability

No new data were created or analyzed in this study. Data sharing is not applicable to this article.
